# Transdiagnostic Assessment of Mental Health and Sleep Registry: Protocol for a Cross-Sectional Study

**DOI:** 10.31083/AP51115

**Published:** 2026-06-30

**Authors:** Azusa Ishii, Kei Muroi, Hiroku Noma, Makoto Nishimura, Shio Maeda, Kaichiro Furutani, Minori Machida, Sumin Lee, Hitomi Oi, Mayu Koike, Aiko Eto, Hui Huang, Shun Nakajima

**Affiliations:** ^1^International Institute for Integrative Sleep Medicine (WPI-IIIS), Tsukuba Institute for Advanced Research (TIAR), University of Tsukuba, 305-8577 Tsukuba, Ibaraki, Japan; ^2^Master's and Doctoral Program in Psychology Graduate School of Comprehensive Human Sciences, University of Tsukuba, 305-8577 Tsukuba, Ibaraki, Japan; ^3^Department of Nursing, Faculty of Nursing, Social Work and Rehabilitation Science, Kyoto Koka University, 615-0882 Kyoto, Kyoto, Japan; ^4^Faculty of Informatics, Kansai University, 569-1095 Takatsuki, Osaka, Japan; ^5^Graduate School of Technology, Industrial and Social Sciences, Tokushima University, 770-8502 Tokushima, Tokushima, Japan; ^6^Psychology Program, Graduate School of Humanities and Social Sciences, Hiroshima University, 739-8524 Higashihiroshima, Hiroshima, Japan; ^7^Psychology Program, School of Education, Hiroshima University, 739-8524 Higashihiroshima, Hiroshima, Japan; ^8^Department of Psychological Sciences, University of Human Environments, 790-0825 Matsuyama, Ehime, Japan; ^9^School of Engineering, Institute of Science Tokyo, 152-8552 Meguro, Tokyo, Japan; ^10^Research Center for Child Mental Development, Chiba University, 263-8522 Chiba, Chiba, Japan; ^11^Department of Sleep Medicine, Affiliated Wuhan Mental Health Center, Tongji Medical College of Huazhong University of Science and Technology, 430000 Wuhan, Hubei, China; ^12^Institute of Medicine, University of Tsukuba, 305-8577 Tsukuba, Ibaraki, Japan

**Keywords:** patient-reported outcomes, registry, mental health, transdiagnostic assessment, digital health, cross-sectional study

## Abstract

**Background:**

Mental disorders impose a substantial burden on individuals and society that cannot be fully captured by diagnostic labels or clinial indicators alone. Accordingly, the need for approaches that comprehensively assess patients’ subjective experiences and daily functioning is growing. Patient-reported outcomes (PROs) enable multidimensional assessment from the patient’s perspective and are increasingly important in mental health research. However, prior studies have frequently assessed these domains in isolation using different instruments and assessment schedules, limiting individual-level integration and cross-study comparability. The focus on single diagnostic groups has further impeded transdiagnostic understanding, and the lack of harmonized data standards has restricted data sharing and reuse. This study protocol describes the establishment of the Transdiagnostic Assessment of Mental Health and Sleep (TAMS) registry, a multidimensional PROs registry designed to enable transdiagnostic comparisons across individuals with mental disorders and physical conditions, as well as healthy participants.

**Methods and Analysis:**

We will conduct a nationwide registry-based cross-sectional study in Japan. Participants will include adults with mental disorders, those with physical conditions, and healthy participants, recruited through an online survey company. After providing electronic consent, all participants will complete an online survey providing information on demographics, diagnoses, and treatment. The registry will also capture information on Quality of Life (QOL) as well as related psychosocial factors, psychiatric symptoms, sleep characteristics, and attitudes toward digital health technology. Data collection is planned for March 2026, with a target enrollment of 3300 participants. Planned analyses will validate patient-reported instruments and characterize how these variables are related to QOL.

**Ethics and Dissemination:**

This study has received ethical approval (ID R07-137) and will be conducted in accordance with national information security guidelines, with voluntary participation, digital informed consent, and findings will be disseminated through peer-reviewed publications and plain-language summaries. The registry will be released as a publicly accessible dataset in accordance with the data-sharing guidelines of the FAIR principles (Findable, Accessible, Interoperable, and Reusable).

**Clinical Trial Registration:**

The study has been registered on https://jrct.mhlw.go.jp/ (registration number: jRCT1030250737; registration link: https://jrct.mhlw.go.jp/latest-detail/jRCT1030250737).

## Main Points

1. This study establishes a multidimensional patient-reported outcomes registry that includes not only individuals with mental disorders and physical conditions but also healthy participants.

2. By moving beyond conventional disorder-specific registries, the registry enables systematic comparison across participants, facilitating transdiagnostic analyses.

3. The registry is designed in compliance with the Findable, Accessible, Interoperable, and Reusable (FAIR) principles, ensuring data findability, accessibility, interoperability, and reusability.

## 1. Introduction

Mental disorders substantially diminish individuals’ quality of life (QOL) and impose high societal costs through losses in educational and employment opportunities, impaired interpersonal relationships, and reduced social participation [1]. The economic impact of mental disorders, including rising health care costs and productivity losses, has also attracted international attention [2], highlighting the need for rigorous evidence on the effectiveness and cost-effectiveness of mental health interventions [2,3]. However, treatment outcomes in mental health care are not adequately captured by symptom reduction alone [4]; comprehensive evaluation requires the explicit incorporation of patients’ subjective experiences, functional changes, and broader psychosocial dimensions [4,5]. Patient-reported outcomes (PROs) address this need by providing a standardized and scalable means of capturing patients’ own perspectives of their symptoms and health-related QOL, which can complement clinician-rated measures [6].

Evidence suggests that integrating PROs into clinical care improves outcomes and supports shared decision-making and quality improvement [7,8,9,10]. Nevertheless, the collection of data on PROs in mental health remains fragmented because of the use of different instruments, inconsistent assessment schedules, and misaligned data standards, resulting in siloed datasets and limited comparability [11]. A health registry directly addresses this fragmentation by providing a structured system for the collection, storage, and dissemination of standardized data from defined populations [12]. As health registries use harmonized instruments and aligned assessment schedules, they enable both individual-level integration and cross-study comparability. However, despite the inception of international initiatives such as Australia’s Mental Health National Outcomes and Casemix Collection and the OECD’s Patient-Reported Indicator Surveys [13,14], dedicated research registries for PROs in mental health remain scarce.

Impairments in functioning and health-related QOL are not confined to specific diagnostic categories within mental disorders but are also observed, to varying degrees, in the general population and among individuals with physical conditions [15,16,17]. Given the frequent comorbidity and temporal dynamics of psychopathological conditions, mental burden is also better conceptualized using continuous dimensions spanning multiple symptom domains than discrete diagnostic categories [18]. Accordingly, transdiagnostic multidimensional frameworks such as the General Psychopathology Factor and Hierarchical Taxonomy of Psychopathology have been proposed [19,20]. However, the data available in most mental health registries limit transdiagnostic analyses and the interpretation of outcome measures, as they focus exclusively on individuals with mental disorders and are organized by diagnosis.

Because transdiagnostic research relies on the linkage, comparison, and reanalysis of data from a variety of studies and populations, a unified framework that explicitly meets these requirements is essential [21]. To enable the linkage, comparison, and reanalysis of PROs data across studies and domains, registries must be designed to support reuse and interoperability as well as adhere to the FAIR principles of Findable, Accessible, Interoperable, and Reusable [22], which extend beyond data availability by emphasizing data structures and metadata that anticipate future reuse and networked integration. Despite the growing number of PROs registries, relatively few explicitly implement the FAIR principles in the design stage or transparently document their level of compliance. The introduction of a transdiagnostic PROs registry guided by these principles could facilitate the integrated assessment of core domains that determine patients’ QOL and functioning within a unified framework.

Any new PROs registry should focus on both patients’ health-related QOL as well as psychosocial factors [23,24,25,26]. In addition, it would be important to account for patients’ psychiatric symptoms, sleep characteristics, and attitudes toward digital health technology. First, sleep disturbance is prevalent across a wide range of mental disorders, including depression, anxiety disorders, and developmental disabilities, as well as in physical illnesses such as diabetes, which exemplifies a condition at the intersection of psychiatric symptoms and QOL impairment [27,28,29,30]. Second, recent advances in digital technologies have accelerated the adoption of remote psychotherapy and artificial intelligence (AI)-based support tools for addressing mental health concerns [31,32]; however, their effectiveness and real-world implementation are strongly influenced by psychosocial factors, including users’ and professionals’ attitudes, ethical concerns, and risk perceptions [31,33]. Importantly, sleep characteristics and attitudes toward digital health technology are not independent domains: sleep is a major target of digital monitoring and intervention, and the uptake and sustained use of these technologies depend on users’ digital literacy, trust, and attitudes. Assessing these domains concurrently within a harmonized registry therefore enables participant-level cross-domain analyses that capture the interface between psychopathology and digital health—analyses that are not easily reconstructed from separate studies.

Despite the relevance of these domains, prior studies have largely relied on domain-specific assessment approaches, constraining the reconstruction of multidimensional, patient-level profiles and the examination of cross-domain patterns spanning psychiatric symptoms, sleep characteristics, and attitudes toward digital health technology. Further, studies that have simultaneously collected these domains within a single, harmonized PROs registry using standardized measurement schedules and data structures remain scarce. This study bridges an important gap in research on this topic because it aims to establish the Transdiagnostic Assessment of Mental Health and Sleep (TAMS) registry in Japan. The TAMS registry intends to provide a standardized infrastructure for the multidimensional collection and unified assessment of QOL, related psychosocial factors, psychiatric symptoms, sleep characteristics, and attitudes toward digital health technology. In this study, a transdiagnostic perspective is adopted as a design requirement for the registry, rather than as a theoretical hypothesis to be tested.

## 2. Materials and Methods

### 2.1 Design

**Fig. 1.**
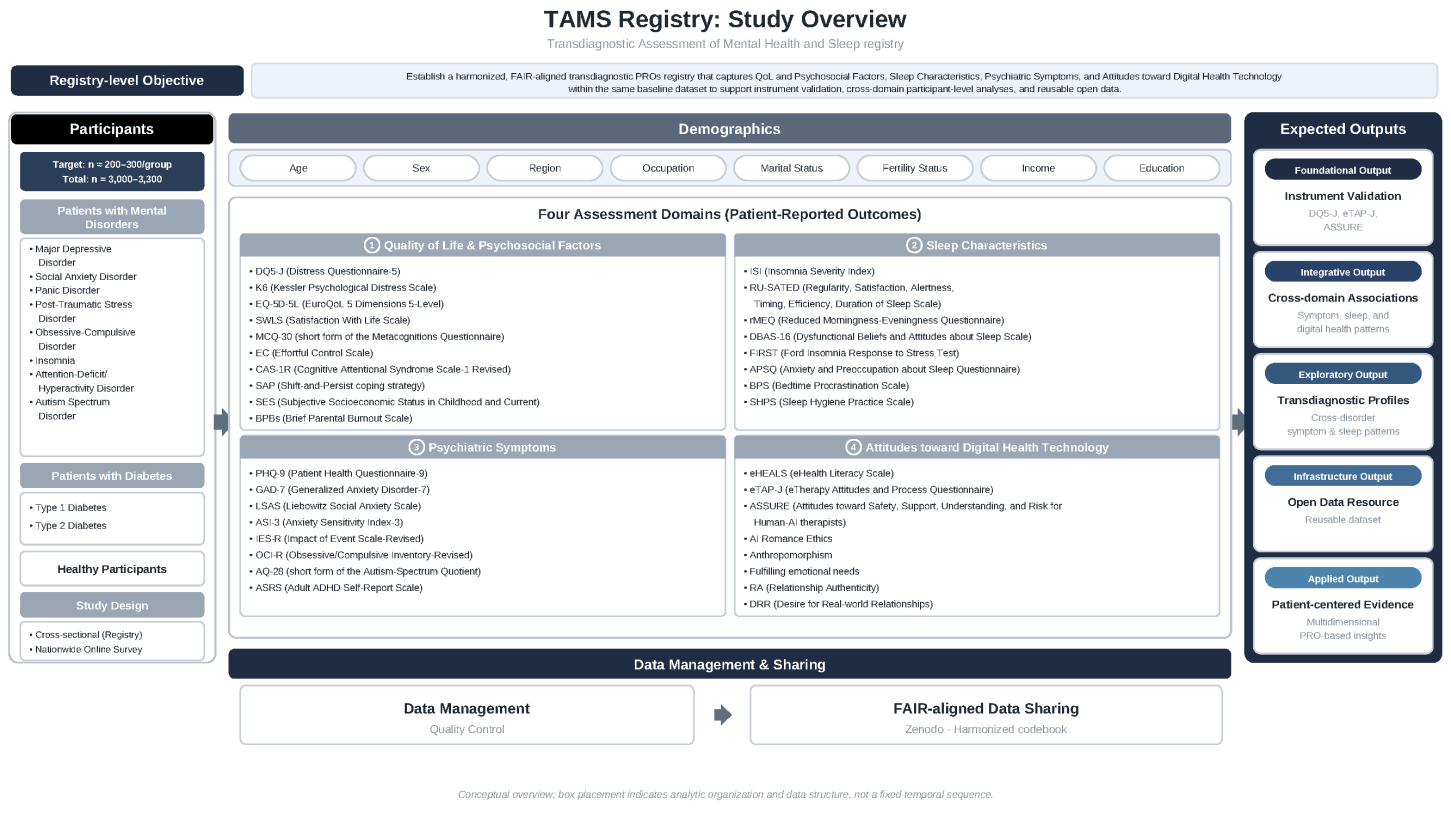
**Study overview: TAMS registry framework**. TAMS, Transdiagnostic Assessment of Mental Health and Sleep registry; FAIR, Findable, Accessible, Interoperable, and Reusable.

The TAMS registry will systematically collect multidimensional data from individuals with mental disorders and physical conditions, as well as from healthy participants in Japan. This proposed framework is expected to support both the psychometric validation of key measures and the exploratory and confirmatory factor analyses (exploratory factor analysis [EFA] and confirmatory factor analysis [CFA], respectively) of the relationships among the clinical, psychosocial, and attitudinal domains. Fig. 1 depicts the registry-level objective, conceptual design, and common data structure of the TAMS registry; it does not intend to represent a fixed temporal or analytic sequence.

Reporting will adhere to the Strengthening the Reporting of Observational Studies in Epidemiology (STROBE) guidelines for observational research [34]. Registry planning and governance will be based on the Agency for Healthcare Research and Quality guidelines [35], Checklist for Reporting Results of Internet E-Surveys (CHERRIES) [36], and Standardized Protocol Items Recommendations for Observational Studies (SPIROS 2025) checklist [37]. In addition, the open-science practices of the registry will conform to the FAIR principles [21] (see **Supplementary Materials**).

### 2.2 Participants

Participants will be recruited in March 2026 from a nationwide web-based research panel managed by Macromill, Inc. (Tokyo, Japan). Recruitment will be conducted through direct invitations and study announcements posted on the Macromill website. The Macromill panel has been used in several previous studies [38,39]. Participants will be recruited using prespecified eligibility criteria and stratified quotas by diagnostic category. The provider maintains both general-population and disease-specific subpanels, including pre-identified panelists who have received physician diagnoses of the target disorders at medical institutions. Diagnostic classification will be based on the participant-reported history of physician diagnosis recorded in Macromill’s disease-specific panels, in which disease information is updated annually. By way of confirmation, invitees will additionally complete a screening item upon starting the survey to confirm their current diagnosis; diagnoses will not be independently verified through medical records or structured diagnostic interviews. Only respondents who pass their confirmation stage will proceed to the survey.

Inclusion criteria will be as follows: (1) understanding the study purpose and providing informed consent; (2) registration in a pre-identified disease-specific panel comprising individuals who have received a physician diagnosis of a target disorder and a positive response to an upfront screening item confirming a current diagnosis of one of the following conditions: major depressive disorder, social anxiety disorder, panic disorder, post-traumatic stress disorder, obsessive/compulsive disorder, insomnia, attention-deficit/hyperactivity disorder, autism spectrum disorder, type 1 diabetes, and type 2 diabetes; or registration as a healthy individual based on self-report indicating no current physician-diagnosed disease; (3) age ≥18 years; and (4) ability to respond in Japanese. Exclusion criteria will include the inability to provide informed consent or complete the survey.

### 2.3 Procedures

All procedures, including registration, consent, survey completion, and a two-week retest, administered to approximately 140 participants with mental disorders to evaluate the test–retest reliability of the five-item Japanese version of the Distress Questionnaire (DQ5-J), will be conducted online. Among retest completers, participants who report “no change” on the Patient Global Impression of Change at the two-week retest will be retained for the primary test–retest reliability analysis. Participation in the survey will be voluntary. Participants who complete the survey will receive points equivalent to 200 yen (approximately US $ 1.30) and those who complete the retest will receive an additional 100-yen worth of points (approximately US $ 0.65).

### 2.4 Measures

The registry will incorporate standardized assessments of QOL and related psychosocial factors, psychiatric symptoms, sleep characteristics, and attitudes toward digital health technology. The following measures will be used. To measure QOL and related psychosocial factors, we will use the DQ5-J, six-item Kessler Psychological Distress Scale (K6) [40], EuroQoL 5 Dimensions 5-Level (EQ-5D-5L) [41,42], Satisfaction With Life Scale (SWLS), short form of the Metacognitions Questionnaire-Short Form (MCQ-30; only the positive beliefs about worry and the negative beliefs about uncontrollability and danger of worry subscales) [43,44], Effortful Control Scale (EC; only attention control subscale) [45,46], Cognitive Attentional Syndrome Scale-1 Revised (CAS-1R; only the coping strategies subscale) [47,48], Shift-and-Persist coping strategy (SAP) [49,50], subjective Socioeconomic Status (SES; childhood and current) [23,24,25], and the Brief Parental Burnout Scale (BPBs) [26]. To measure sleep characteristics, we will use the Insomnia Severity Index (ISI) [51,52], Regularity, Satisfaction, Alertness, Timing, Efficiency, Duration of Sleep Scale (RU-SATED) [53,54], Reduced Morningness–Eveningness Questionnaire (rMEQ) [55,56], Dysfunctional Beliefs and Attitudes about Sleep Scale (DBAS-16) [57,58], Ford Insomnia Response to Stress Test (FIRST) [59,60], Anxiety and Preoccupation about Sleep Questionnaire (APSQ) [61,62], Bedtime Procrastination Scale (BPS) [63,64], and Sleep Hygiene Practice Scale (SHPS) [65,66]. To measure psychiatric symptoms, we will use the Patient Health Questionnaire-9 (PHQ-9) [67,68], Generalized Anxiety Disorder-7 (GAD-7) [69,70], Liebowitz Social Anxiety Scale (LSAS) [71,72], Anxiety Sensitivity Index-3 (ASI-3) [38,73], Impact of Event Scale-Revised (IES-R) [74,75], Obsessive/Compulsive Inventory-Revised (OCI-R) [76,77], short form of the Autism-Spectrum Quotient (AQ-28) [78], and Adult ADHD Self-Report Scale (ASRS) [79]. To measure attitudes toward digital health technology, we will use the eHealth Literacy Scale (eHEALS) [80,81], Japanese version of the e-Therapy Attitudes and Process Questionnaire (eTAP-J) [82], and Attitudes toward Safety, Support, Understanding, and Risk for Human-AI therapists (ASSURE; two parallel scales: ASSURE-HT and ASSURE-AI), AI romance ethics, Anthropomorphism [83], Fulfilling emotional needs [84,85], Relationship Authenticity (RA) [86], and Desire for Real-World Relationships (DRR) [86]. Additionally, we will collect basic demographic characteristics, including age, sex, region of residence, occupation, marital status, fertility status, income status, and educational level. The survey consists of 1–40 items per page across 45 pages, and all items are mandatory.

### 2.5 Study Aims and Analytic Strategy

Table 1 summarizes the three aims, sub-studies, outcomes, and planned analyses; the full analytic specifications are provided in the **Supplementary Materials**. Consistent with the AHRQ User’s Guide for Patient Registries, which notes that a registry may serve multiple stated purposes [12] but that its overall purpose should be translated into specific objectives or research questions, no single clinical primary outcome is defined for the registry as a whole; rather, the registry-level objective is to establish a harmonized transdiagnostic PROs infrastructure. Therefore, each sub-study has its own analytic objective, study population, key variables, and key outcomes (Table 1). Conceptually, the sub-studies are organized into foundational psychometric validation, hypothesis-driven association analyses, and exploratory profiling analyses; this grouping reflects their analytic role rather than a temporal order. The distinction between confirmatory and exploratory analyses is explicitly indicated in the “Analysis type” column of Table 1. Confirmatory analyses are based on prespecified hypotheses, whereas exploratory analyses are hypothesis-generating in nature. Therefore, findings from exploratory analyses should be interpreted with caution. Replication must be conducted in future studies before firm conclusions can be drawn.

**Table 1.** **Overview of the study aims, populations, objectives, key variables, analysis plans, and analysis types**.

Study aim/Sub-study	Population	Objective/Hypothesis	Key variables	Analysis plan	Analysis type
**Aim 1. Psychosocial correlations and measurement foundations for QOL and psychiatric symptoms**					
1a. DQ5-J validation	Patients with mental disorders	Examine the reliability and validity of the DQ5-J.	Target instrument: DQ5-J Validation measures: PHQ-9, GAD-7, EQ-5D-5L	Structural validity: CFA first, EFA only if needed Reliability: Internal consistency (*α*, *ω*), Test–retest (intraclass correlation coefficient) Construct validity: Convergent/discriminant/known-groups validity testing Criterion validity: receiver operating characteristic/area under the curve analysis	Confirmatory
1b. Parental burnout in relation to socioeconomic status, mental health, and sleep	Patients with mental disorders	Examine the associations of BPBs with current SES, psychiatric symptoms, neurodevelopmental traits, sleep measures, and QOL.	Exposure: BPBs Correlates/outcomes: current SES, psychiatric symptoms (e.g., PHQ-9, GAD-7, K6), neurodevelopmental traits (e.g., AQ-28, ASRS), sleep measures (e.g., ISI, RU-SATED), and QOL (e.g., SWLS and EQ-5D-5L)	Pearson/Spearman correlations Partial correlations adjusting for age, sex, and prespecified demographics FDR correction (Benjamini–Hochberg procedure, *q* < 0.05) Outlier identification and sensitivity analyses	Confirmatory
1c. Shift-and-persist coping strategies in relation to mental disorders	Patients with mental disorders, healthy participants	Examine whether individual differences in SAP coping strategies are associated with case–control status across multiple mental disorders and whether higher SAP are associated with a lower likelihood of belonging to patient groups than a shared non-clinical control group.	Exposure: Shift-and-persist coping strategies (quartiles; continuous in sensitivity analyses) Outcomes: case–control status within each mental disorder panel (patient vs non-clinical control) Covariates: age, sex, education, SES (childhood and current)	Chi-square tests, logistic regression (odds ratios, 95% confidence intervals), Cochran–Armitage trend tests, Firth-penalized logistic regression, FDR correction (Benjamini–Hochberg procedure, *q *< 0.05), sensitivity analyses	Confirmatory
1d. Metacognitive beliefs, CAS, and attentional control in relation to QOL and psychiatric symptoms	Patients with mental disorders, healthy participants	Examine whether metacognitive beliefs are associated with QOL via CAS, moderated by attention control, and identify CAS coping profiles.	Predictor: MCQ-30 (positive beliefs about worry and the negative beliefs about uncontrollability and danger of worry subscales) Mediator: CAS-1R (coping strategies subscale) Moderator: EC (attention control subscale) Outcomes: EQ-5D-5L (all groups); disorder-specific symptom severity (secondary outcomes)	Multi-group SEM (moderated mediation) LPA on the coping strategies subscale of the CAS-1R (six items): maximum likelihood with robust standard errors, model selection by the Bayesian information criterion, entropy, class interpretability Bolck–Croon–Hagenaars method (continuous outcomes)/DU3STEP (categorical outcomes) EQ-5D-5L minimal important difference-based deterioration (exploratory) Likelihood ratio tests, model fit indices comparison Supplementary LPA on MCQ-30 and EC Sensitivity analyses	Mixed
**Aim 2. Sleep characteristics and psychiatric symptom profiles**					
2a. Sleep profiles in patients with mental disorders	Patients with mental disorders	Identify latent profiles integrating sleep symptoms.	Clustering features: Sleep measures (e.g., ISI, RU-SATED), External validators: EQ-5D-5L, SWLS	Dimension reduction: Uniform manifold approximation and projection Clustering: Density-Based Spatial Clustering of Applications with Noise, hierarchical clustering, k-means, Gaussian mixture models Group comparisons across profiles: one-way ANOVA with Tukey’s HSD (if assumptions met) or Kruskal–Wallis test with Steel–Dwass post-hoc tests for continuous variables; chi-square tests for categorical variables.	Exploratory
2b. Sleep profiles in patients with diabetes	Type 1 and type 2 diabetes	Identify latent profiles integrating sleep symptoms.	Clustering features: same as 2a External validators: EQ-5D-5L, SWLS	Same as 2a, plus between-group comparisons of profile distributions and validators	Exploratory
2c. Psychiatric profiles in patients with developmental disabilities	Patients with developmental disabilities	Identify latent profiles integrating psychiatric symptoms.	Clustering features: Psychiatric symptoms (e.g., PHQ-9, GAD-7) External validators: EQ-5D-5L, SWLS	Same as 2a	Exploratory
**Aim 3. Attitudes toward digital health technology**					
3a. eTAP-J validation	Patients with mental disorders, healthy participants	Examine the reliability and validity of the Japanese version of the eTAP.	Target instrument: eTAP-J Validation measures: eHEALS	Structural validity: CFA Convergent and discriminant validity: correlation Factors influencing the intention to use psychological support delivered via digital technologies: mediation analysis or SEM	Confirmatory
3b. ASSURE-HT, ASSURE-AI validation	Patients with mental disorders	Examine the reliability and validity of ASSURE-HT and ASSURE-AI.	Target instruments: ASSURE-HT (15 items) and ASSURE-AI (15 items) Validation measures: prior AI/face-to-face therapy experience	Structural validity: CFA Internal consistency: Cronbach’s *α* and *ω* (evaluated separately for ASSURE-HT and ASSURE-AI) Known-groups validity: score differences by prior treatment/therapy experience (ASSURE-HT and ASSURE-AI analyzed separately) Differences in attitudes by demographic variables (age group, sex): ANOVA or regression; Chi-square test or ordinal logistic regression (depending on outcome scale type)	Confirmatory
3c. Ethical concerns about romantic relationships with AI	Patients with mental disorders	Examine to what extent patients with mental disorders consider ethical concerns about romantic relationships with AI.	Predictor: anthropomorphism Mediators: relationship authenticity, desire for real-world relationships with AI Outcome: fulfilment of emotional needs	Correlations, serial mediation	Confirmatory

Abbreviations: ANOVA, Analysis of variance; CFA, Confirmatory factor analysis; DU3STEP, Three-step approach for distal outcomes in latent class/profile analysis; EFA, Exploratory factor analysis; FDR, False discovery rate; LPA, Latent profile analysis; SEM, Structural equation modeling; *α*, Cronbach’s alpha; *ω*, McDonald’s omega; QOL, uality of Life; DQ5-J, Distress Questionnaire; PHQ-9, Patient Health Questionnaire-9; GAD-7, Generalized Anxiety Disorder-7; EQ-5D-5L, EuroQoL 5 Dimensions 5-Level; BPBs, Brief Parental Burnout Scale; SES, Subjective Socioeconomic Status (Childhood and Current); K6, six-item Kessler Psychological Distress Scale; AQ-28, Autism Spectrum Quotient; ASRS, Adult ADHD Self-Report Scale; ISI, Insomnia Severity Index; RU-SATED, Regularity, Satisfaction, Alertness, Timing, Efficiency, Duration of Sleep Scale; SWLS, Satisfaction With Life Scale; SAP, Shift-and-Persist coping strategy; MCQ-30, Metacognitions Questionnaire; CAS-1R, Cognitive Attentional Syndrome Scale-1 Revised; EC, Effortful Control Scale; HSD, Honestly Significant Difference; eTAP-J, Japanese version of the e-Therapy Attitudes and Process Questionnaire; eHEALS, eHealth Literacy Scale; ASSURE-HT and ASSURE-AI, Attitudes toward Safety, Support, Understanding, and Risk for Human-AI therapists; AI, artificial intelligence.

#### 2.5.1 Aim 1: Psychosocial Correlations and Measurement Foundations for QOL and Psychiatric Symptoms

The registry will examine the reliability and validity of the DQ5-J and conduct cross-sectional association analyses to investigate how BPBs, SAP, SES, and metacognitive beliefs and coping strategies (MCQ-30 and CAS-1R) are associated with the QOL and psychiatric symptoms measures. For the analyses, we will employ standard correlational and multivariable modeling approaches as well as perform the exploratory profiling of coping strategies.

#### 2.5.2 Aim 2: Sleep Characteristics and Psychiatric Symptom Profiles

The registry will identify data-driven profiles integrating the sleep characteristics and/or psychiatric symptoms measures in individuals with mental disorders and examine their associations with QOL and symptom severity. The same profiling pipeline will be applied to participants with type 1 and type 2 diabetes as well as those with developmental disabilities.

#### 2.5.3 Aim 3: Attitudes Toward Digital Health Technology

The registry will evaluate the measurement properties of the instruments assessing attitudes toward and ethical concerns about digital health technology in the mental health domain, including the eTAP-J and ASSURE scales (ASSURE-HT, ASSURE-AI). In addition, ethical concerns about romantic relationships with AI will be examined given their potential influence on the acceptance of and engagement with emerging digital health interventions.

### 2.6 Sample Size and Sampling

Because this is a registry-based observational study, the study size will primarily be determined by the inclusion of as many feasible participants as possible within prespecified diagnostic strata, consistent with the survey company’s prior recruitment performance. A pragmatic target of 200–300 participants per group will be set, yielding an overall enrollment target of 3000–3300 participants. To contextualize this range, 200–300 participants per stratum corresponds approximately to 95% confidence-interval half-widths of ± 6.9% to ± 5.7% for proportions near 0.50, detectable standardized mean differences of *d *≈ 0.28 to 0.23 for two-group comparisons, and detectable correlations of *r *≈ 0.20 to 0.16. For more complex structural equation modeling and person-centered analyses, adequacy depends strongly on the model complexity, measurement quality, class separation, convergence, and stability; accordingly, these analyses will be interpreted primarily using model fit, convergence, and stability criteria, and disease-specific profiling analyses will be undertaken only if sufficient sample sizes are obtained [34].

Because online surveys tend to disproportionately attract younger respondents, age-stratified quota sampling will be employed to ensure adequate representation across age groups. Minimum quotas will be set at 20 participants per group for each age decade in the 20s (including ages 18 and 19), 30s, 40s, and 50s, and at least 10 participants per group for individuals aged 60 years and older. These quotas will be applied flexibly in groups with limited numbers of registered panel members.

### 2.7 Statistical Analysis

To minimize information bias, we will conduct logic and range checks during data cleaning. Descriptive analyses will summarize distributions, floor and ceiling effects, and reliability indices. Unless otherwise specified, analyses will use two-sided tests, apply pre-specified multiple comparison correction strategies that differ by sub-study (see **Supplementary Materials**), and control for prespecified covariates (e.g., age and sex). Prespecified subgroup comparisons and corresponding interaction tests for diagnostic strata and sex will be conducted a priori, whereas other interaction analyses will be considered exploratory. Effect sizes with 95% confidence intervals will be reported, including Cohen’s *d *and Hedges’ *g* for continuous outcomes, odds ratios from logistic regression models, and standardized *β* coefficients for regression paths. Because the panel provider supplies only complete records for both the survey and the two-week retest, analyses will be restricted to complete-case data. Additional methodological details are provided in the **Supplementary Materials**.

In Sub-study 1a of Aim 1, all the analyses will be conducted in accordance with the Consensus-based Standards for the selection of health Measurement Instruments (COSMIN) guidelines [87]. The psychometric validation of the DQ5-J will include CFA to test the a priori unidimensional structure, with EFA reserved as a follow-up if the model fit is inadequate, internal consistency indices (Cronbach’s alpha and McDonald’s omega), test–retest intraclass correlation coefficients, model fit criteria, hypothesis-driven convergent and discriminant validity testing, and receiver operating characteristic analyses.

In Sub-studies 1b–1d of Aim 1, the correlations between the psychosocial factors and QOL/psychiatric symptoms will be examined by assessing the associations among the BPBs scores, SAP, SES, the psychiatric symptoms and sleep characteristics measures, and QOL using correlation analyses, linear regression models, and logistic regression. Sub-study 1d will additionally apply multiple-group structural equation modeling with moderated mediation and latent profile analysis to characterize the coping strategies assessed by the CAS-1R [88,89,90].

For Aim 2, latent profiles integrating the sleep characteristics and psychiatric symptoms measures will be derived using uniform manifold approximation and projection embeddings in combination with multiple clustering algorithms. The optimal number of clusters will be determined according to a comprehensive clustering validation framework [91], incorporating multiple internal validation criteria. Differences in the psychological and demographic variables between the clusters will be evaluated using one-way analysis of variance (ANOVA) with Tukey’s Honestly Significant Difference (HSD) post-hoc test when assumptions are met, or the Kruskal–Wallis test with Steel–Dwass post-hoc comparisons otherwise, for continuous variables and chi-square tests for categorical variables.

For Aim 3, the analyses of measurement foundations and attitudes toward digital health technology and AI therapy will include CFA for the eTAP-J, ASSURE-HT, and ASSURE-AI scales as well as regression and ANOVA models to assess group differences. The associations among attitudes toward digital health technology (e.g., acceptance, anthropomorphism, and ethical concerns) and QOL will be examined using serial mediation models.

To assess the robustness of the main findings, prespecified sensitivity analyses will be conducted by varying the model specifications and key assumptions. Specifically, the analyses will be re-estimated using alternative scoring approaches or short-form versions of the questionnaires, varying the thresholds for categorized outcomes, allowing non-linear terms for continuous predictors, and perturbing clustering parameters. The stability of the results will be evaluated across these alternative specifications.

### 2.8 Data Management and Quality Assurance

Data collection will be conducted through Macromill’s secure online research platform. At the beginning of the survey, participants will be presented with a pledge item asking them to commit to responding carefully, and only those who agree will be allowed to proceed to the subsequent questions [92]. The research team will receive only anonymized survey data from Macromill; no directly identifiable personal information (e.g., names, addresses, e-mail addresses, and contact details) will be provided. According to the company’s publicly available quality management policy, duplicate registrations using the same e-mail address or overlapping personal information are automatically rejected at the system level. Furthermore, during survey administration, the system identifies device cookies to prevent multiple submissions from the same terminal. Consequently, duplicate responses from the same individual are technically prevented. The survey interface requires the completion of all mandatory items and incorporates built-in logic and consistency checks to ensure data integrity. To enhance response quality, the survey platform automatically saves responses upon page transition, allowing participants to pause and resume at any point. To protect data validity, completion within 24-hours of survey initiation is required; surveys not submitted within this window are treated as withdrawal of consent and excluded from analysis.

Files are transferred via institution-approved encrypted channels and stored on access-controlled servers. According to the FAIR principles [21], a prespecified data dictionary governs variable naming conventions, valid ranges, skip patterns, scoring rules, and derivations. Using this specification, the research team generates a de-identified analytic dataset and a versioned codebook with an auditable change log. Following the publication of the primary results, the registry dataset will be released through Zenodo, with a dataset-level DOI assigned at publication. Record metadata will be available in machine-readable formats, including DataCite, DCAT, and JSON-LD using Schema.org vocabulary. A public versioned data dictionary will accompany each release in CSV format, with clearly labeled columns and definitions documented in an accompanying README. Programmatic access will be supported through Zenodo’s REST API and OAI-PMH; no custom API and variable-level persistent identifiers are planned for the initial release. Each release will also include clear licensing, versioning, and provenance documentation to support reuse. Additional details on governance, data derivation and versioning, access control, data retention and disposal, and dataset separation are provided in the **Supplementary Materials**.

### 2.9 Ethics and Dissemination

The study protocol has received ethical approval from the relevant institutional review board (ID R07-137). Digital informed consent will be obtained from all participants before participation. The study findings will be disseminated through peer-reviewed publications, conference presentations, and plain-language summaries for participants and patient advocacy organizations.

The study has been registered in the Japan Registry of Clinical Trials (registration number: jRCT1030250737; registration link: https://jrct.mhlw.go.jp/latest-detail/jRCT1030250737; February 17, 2026). The registration includes the details of the study objectives, design, and data management procedures. Any protocol amendments will be reflected in updates to the records of the Japan Registry of Clinical Trials.

Participation in the study may involve temporary discomfort or psychological burden. Completion of the self-administered questionnaires (approximately 110 minutes in total) may impose a time burden on participants. Although no directly identifiable personal information will be collected, the risk of data breaches or unauthorized access cannot be eliminated.

To mitigate these risks, survey items have been restricted to the minimum necessary to achieve the study objectives. All research procedures will comply with national and institutional information security guidelines. Only devices with up-to-date security software will be used; temporary cloud-stored data will be promptly deleted; and all data will be stored in encrypted form and accessed exclusively by authorized researchers.

Participants who experience psychological distress during or after the study may contact the survey company’s consultation service or the research team directly. Participation will be voluntary, and participants may withdraw at any time. Responses will be analyzed only if the survey is completed and formally submitted.

## 3. Discussion

This study establishes the TAMS registry as a foundational resource for the transdiagnostic evaluation of QOL and related psychosocial factors, psychiatric symptoms, sleep characteristics, and attitudes toward digital health technology across individuals with mental disorders and physical conditions as well as healthy participants. The TAMS registry represents a first step toward addressing the longstanding fragmentation of mental health research by enabling the concurrent assessment of these domains within a unified framework. By adopting a registry design aligned with the FAIR principles, this infrastructure is intended to support data sharing, secondary analyses, and cumulative knowledge generation beyond individual studies.

More broadly, the significance of the TAMS registry lies in its potential to reshape how mental health outcomes are studied and interpreted. Prior research has frequently examined psychiatric symptoms, sleep characteristics, and psychosocial factors in isolation, limiting the reconstruction of multidimensional, patient-level profiles and the investigation of cross-domain relationships. By providing a unified framework across diagnostic groups, the registry enables the exploration of shared and disorder-specific patterns of psychosocial factors and sleep characteristics, thereby supporting transdiagnostic profiling and hypothesis generation rather than prespecified causal inference.

The inclusion of multiple diagnostic groups within a single registry further advances this objective by enabling direct comparisons across patients with mental disorders and physical conditions as well as healthy participants, using a common measurement framework. This design facilitates the identification of dimensions that cut across diagnostic categories while preserving the capacity to examine disorder-specific features. In this respect, the TAMS registry enables future stratified analyses, data-driven subtyping, and comparative research. Importantly, the alignment of the registry with the FAIR principles constitutes a methodological contribution in its own right. Despite the growing number of PROs registries, the explicit implementation of the FAIR principles in the design stage remains limited. By systematizing data structures, metadata, and documentation to anticipate reuse, the TAMS registry lowers barriers to data integration and secondary use, promoting transparency, reproducibility, and international collaboration.

Beyond the inclusion of multiple diagnostic groups and FAIR alignment, the breadth of the instrument set reflects a deliberate design feature balanced against the trade-off of respondent burden. Cross-domain analyses spanning QOL, psychiatric symptoms, sleep characteristics, and attitudes toward digital health technology are difficult to reconstruct from narrower, domain-specific assessment strategies alone, which has been the dominant approach in prior research and a key gap this registry was designed to address. To balance this comprehensiveness against participant burden, the protocol deliberately employs (i) brief or screening-grade instruments where psychometrically defensible (e.g., K6, GAD-7, PHQ-9, DQ5-J, EQ-5D-5L); (ii) selective administration of relevant subscales rather than full inventories where appropriate (e.g., only 2 subscales of the MCQ-30, only the attention-control subscale of the EC, only the coping-strategies subscale of the CAS-1R, and the short form of the AQ); and (iii) operational features that mitigate fatigue during administration (auto-save on page transition, pause-and-resume within a 24-hour window, and a quality-check pledge item; see Section 2.8). We nevertheless acknowledge that respondent burden remains substantial, and the present design should be understood as a deliberate trade-off between analytic breadth and participant burden.

Operationally, the cross-sectional design means nationwide online panel data are collected by a survey company with participant identification assured; however, the analytic dataset contains no directly identifiable personal information. Following publication of the primary results, the data are planned to be released as open data under a CC BY 4.0 license and made available for direct download from a DOI-minting repository. This data-sharing strategy is expected to promote broad secondary use, including reanalysis and comparative research, while maintaining ethical safeguards and transparency, contributing to research reproducibility and the accumulation of international evidence in mental health research.

Some limitations should be noted. First, although the use of a nationwide online panel facilitated rapid enrollment, it could have introduced selection bias and limited broad generalizability [93]. The online panel sample may differ systematically from general clinical populations in terms of health consciousness, digital literacy, and willingness to participate in research [94,95]. Conversely, reliance on pre-registered disease-specific panels may underrepresent individuals with more severe illnesses or lower treatment engagement, potentially biasing the estimates toward milder presentations. Prior evidence suggests that individuals with mental disorders tend to be slightly underrepresented in general population-based surveys [96], and the direction of this selection bias should be considered when interpreting findings.

Second, diagnostic classification relied on participant-reported physician diagnosis, confirmed by a screening item at survey entry; it was not independently verified through medical records or structured diagnostic interviews for any diagnostic group, including the psychiatric disorder and diabetes groups. Research comparing self-reported diagnoses with symptom-based diagnostic methods in large online cohort studies has demonstrated only moderate agreement, with a substantial proportion of individuals with self-reported diagnoses not meeting symptom-based criteria, and vice versa [97]. Diagnostic misclassification of this form is likely to attenuate true between-group differences, potentially leading to an underestimation of associations between diagnostic group membership and the outcomes of interest. For psychiatric disorder groups, analyses restricted to participants whose self-reported diagnosis is corroborated by scores meeting established clinical cutoffs on the corresponding symptom measures may provide supplementary information; however, such analyses would necessarily exclude participants who are in remission at the time of assessment (such as individuals with a valid lifetime diagnosis whose current symptom scores fall below clinical thresholds) and would represent a systematically different subsample from the primary analyses [97]. For the diabetes group, symptom-based corroboration is not feasible as no corresponding symptom severity measures were collected. As a complementary sensitivity analysis, where suitable external estimates of the sensitivity and specificity of participant-reported history of physician diagnosis are available for a given disorder, quantitative bias analysis methods may be explored to assess the potential impact of diagnostic misclassification on selected prevalence or between-group estimates. For prevalence-type quantities, this may include classical correction approaches such as the Rogan–Gladen estimator [98], whereas broader probabilistic bias analysis frameworks may also be considered [99]. However, because valid external sensitivity and specificity parameters are not uniformly available across all disorders included in the registry, this approach is not prespecified as a systematic analysis across all diagnostic strata.

Third, a more fundamental limitation of the cross-sectional design is the inability to distinguish state from trait (e.g., whether poor sleep is a cause or a consequence of depression) and the inability to infer causal directionality in mediation models. All mediation analyses and structural equation modeling (SEM) models employed across the registry should be interpreted with caution, as the relations identified reflect statistical associations only and do not support causal inference. Indeed, cross-sectional mediation analyses yield biased estimates of longitudinal indirect effects [100]. The moderated mediation analyses in Sub-study 1d are subject to the same constraint and can only characterize conditional association patterns, not causal mediation. Therefore, the findings should be interpreted as hypothesis-generating evidence to be examined in future longitudinal designs.

Finally, although several measures were implemented to minimize careless responding before data collection, including a quality-check pledge item and built-in logic and consistency checks, the identification of inattentive responding in clinical populations presents methodological challenges. Commonly used detection indices, such as longstring analysis, Mahalanobis distance, and intra-individual response variability, may be confounded with respondents’ characteristics and genuine response tendencies, making it difficult to determine whether flagged responses reflect genuine inattention or valid patterns of responding [92,101]. Accordingly, the potential presence of residual inattentive responding cannot be ruled out as a source of measurement error in the present data.

Looking ahead, the TAMS registry may provide a foundation for future longitudinal extensions. Subject to funding, ethics approval, and the operational feasibility of panel re-contact through the survey provider, a follow-up wave could be considered using an abbreviated core-PROs battery to reduce respondent burden while preserving the main cross-domain constructs. In addition, FAIR-aligned release of the baseline dataset may facilitate harmonization or comparative use alongside independent longitudinal datasets. Such extensions could help examine temporal ordering more directly, evaluate the stability of transdiagnostic profiles over time, and characterize longitudinal changes in attitudes toward digital health technology.

In summary, the TAMS registry, a PROs registry that integrates a comprehensive set of indicators within a single standardized framework is expected to elucidate both common and disorder-specific structures as well as provide a foundation for advancing psychiatric research and patient-centered care. At the theoretical level, the registry is positioned to support the empirical examination of transdiagnostic dimensional structures [19,20], contributing to the development of more integrated psychopathology models that transcend traditional categorical nosology. At the practical level, registry-derived transdiagnostic profiles may inform measurement-based approaches to clinical assessment [7,8,9,10], support evidence-based health policy and resource allocation, and guide the design of digital technologies in the mental health domain by situating attitudes and ethical concerns within the broader context of clinical outcomes [31,32,33].

## 4. Conclusions

This protocol describes the establishment of a multidimensional PROs registry designed to provide a unified framework for evaluating patient-relevant outcomes in mental health. By enabling the concurrent assessment of QOL and psychosocial factors, psychiatric symptoms, sleep characteristics, and attitudes toward digital health technology using a standardized measurement system, the registry addresses the key limitations of prior research in which these domains have typically been examined in isolation.

## Data Availability

Since this article describes the protocol for the TAMS registry, no individual-level dataset is available at the time of publication. The data will be available from the corresponding author upon reasonable request.

## Abbreviations

APSQ, Anxiety and Preoccupation about Sleep Questionnaire; ASSURE-HT and ASSURE-AI, Attitudes toward Safety, Support, Understanding, and Risk for Human-AI Therapists; AQ-28, short form of the Autism Spectrum Quotient; ASI-3, Anxiety Sensitivity Index-3; ASRS, Adult ADHD Self-Report Scale; BPBs, Brief Parental Burnout Scale; BPS, Bedtime Procrastination Scale; CAS-1R, Cognitive Attentional Syndrome Scale-1 Revised; DBAS-16, Dysfunctional Beliefs and Attitudes about Sleep Scale; DQ5-J, Japanese version of the Distress Questionnaire-5; DRR, Desire for Real-World Relationships; EC, Effortful Control Scale; eHEALS, eHealth Literacy Scale; eTAP-J, Japanese version of the e-Therapy Attitudes and Process Questionnaire; EQ-5D-5L, EuroQol 5 Dimensions 5-Level; FAIR, Findable, Accessible, Interoperable, and Reusable; FIRST, Ford Insomnia Response to Stress Test; GAD-7, Generalized Anxiety Disorder 7-item Scale; IES-R, Impact of Event Scale-Revised; ISI, Insomnia Severity Index; K6, six-item Kessler Psychological Distress Scale; LSAS, Liebowitz Social Anxiety Scale; MCQ-30, short form of the Metacognitions Questionnaire; OCI-R, Obsessive/Compulsive Inventory-Revised; PHQ-9, Patient Health Questionnaire-9; PROs, Patient-Rreported Outcomes; QOL, Quality of Life; RA, Relationship Authenticity; rMEQ, Reduced Morningness–Eveningness Questionnaire; RU-SATED, Regularity, Satisfaction, Alertness, Timing, Efficiency, Duration of Sleep Scale; SAP, Shift-and-Persist coping strategy; SES, Subjective Socioeconomic Status (Childhood and Current); SHPS, Sleep Hygiene Practice Scale; SWLS, Satisfaction With Life Scale.
