# Supplementary material for: Transdiagnostic Assessment of Mental Health and Sleep Registry: Protocol for a Cross-Sectional Study
Source: Alpha Psychiatry. 2026 Jun 30;27(3):51115. doi: 10.31083/AP51115 (PMC13339798; doi:10.31083/AP51115)
Supplement: Supplementary file 1 [file 2757-8038-27-3-51115-s1.zip › Supplementary Material.docx]

**Supplementary Materials**

# Participant Recruitment

Participants will be recruited through an established online survey panel operated by Macromill Inc. Individuals who click the survey link will be directed to the study-specific landing page developed by the research team. In addition, a test–retest subsample of approximately 140 participants will be recruited to complete the DQ5-J again two weeks after baseline to evaluate its test–retest reliability within the translation and validation process.

# Eligibility Criteria

## Online Registry Survey

### Inclusion Criteria

・Understanding the study purpose and providing informed consent.

・Registration as panel monitors involves one of the following diagnoses: major depressive disorder, social anxiety disorder, panic disorder, post-traumatic stress disorder, obsessive/compulsive disorder, insomnia, attention-deficit/hyperactivity disorder, autism spectrum disorder, type 1 diabetes, type 2 diabetes, and healthy participants.

・Age ≥18 years.

・Ability to respond in Japanese.

### Exclusion Criteria

・Failure to endorse a quality-check pledge item.

・Incomplete survey submission.

## Test–Retest Subsample

A subset of participants with mental disorders will be invited to complete the DQ5-J again two weeks after baseline (target analytic *n*=140).

### Inclusion Criteria

・Completion of the baseline survey.

・Willingness to participate in a follow-up survey.

・Availability for recontact within the two-week window.

・Patient Global Impression of Change (PGIC) at two-week retest = “no change.”

### Exclusion Criteria

・Withdrawal of consent.

・Failure to complete the baseline survey in full.

# Informed Consent Procedures

Before accessing the baseline survey, participants will be presented with an informed consent form in Japanese. They will be required to review the form and provide electronic consent before proceeding.

# Measures

## Demographics and Clinical History

In this study, we will collect demographic variables including age, sex, region of residence, occupation, marital status, fertility status, income status, and educational level. Clinical characteristics include history of mental or physical disorders, treatment status, medication use (including hypnotics), and the presence of sleep-related symptoms. In addition, psychosocial factors such as family composition and interpersonal relationships, experience with technology and services, and lifestyle (e.g., sleep patterns, commuting time, and napping) are assessed. All the measures used in this survey are the Japanese versions. The designation “Japanese version” is omitted for all the instruments except for the DQ5 and eTAP, for which Japanese versions are being developed (Supplementary Table 1).

## Quality of Life (QOL) and Related Psychosocial Factors

### Japanese Version of the Distress Questionnaire (DQ5-J, five items)

Brief screening of general psychological distress based on the DSM-5 criteria. Items rated 1–5, total 5 to 25. Higher scores = greater distress. To be validated in this study.

### Kessler Psychological Distress Scale (K6, six items)

Assesses non-specific psychological distress related to depression and anxiety over the past 30 days. Each item is rated on a five-point Likert scale (0=none of the time to 4=all of the time), yielding a total score ranging from 0 to 24, with higher scores indicating greater psychological distress. A cut-off score of ≥13 suggests severe psychological distress. Japanese version validated.

### EuroQol 5 Dimensions 5-Level (EQ-5D-5L)

Assesses health-related QOL across five domains (mobility, self-care, usual activities, pain/discomfort, anxiety/depression). Produces an index score (0 to 1) and visual analog scale. Japanese version validated.

### Satisfaction With Life Scale (SWLS, five items)

Assesses global life satisfaction. Items rated 1 to 7; in total, 5 to 35. Higher scores = greater life satisfaction. Japanese version validated.

### Short Form of the Metacognitions Questionnaire (MCQ-30, only Positive Beliefs About Worry and the Negative Beliefs about Uncontrollability and Danger of Worry Subscales, 12 items)

Assesses trait-level metacognitive beliefs about worry. The original MCQ-30 includes 30 items with a five-factor structure; in this study, only two subscales are used: positive beliefs about worry (six items) and negative beliefs about uncontrollability and danger of worry (six items). Each item is rated on a four-point scale (1=do not agree to 4=agree very much). Higher subscale scores indicate stronger maladaptive metacognitive beliefs about worry. Japanese version validated.

### Effortful Control Scale (EC; only Attention Control Subscale, 12 items)

Measures trait-level attentional control within the broader construct of effortful control. The attention control subscale comprises 12 items rated on a seven-point scale; scores are calculated as the sum or mean of item responses, with higher values indicating stronger attentional control. Japanese version validated.

### Cognitive Attentional Syndrome Scale-1 Revised (CAS-1R, Coping Strategies Subscale, six items)

Assesses the coping strategies associated with cognitive attentional syndrome (CAS). The original CAS-1R includes multiple subscales; however, in this study, only the coping strategies subscale (six items) is used. Each item is rated on an 11-point scale (0=none of the time to 100=all of the time, in increments of 10). Higher scores indicate more frequent use of CAS-related coping strategies. Japanese version validated.

### Shift-and-Persist coping strategy (SAP, 14 items)

Assesses adaptive coping strategies (shifting and persisting subscales). Items rated 1 to 6, higher scores = stronger coping strategy. Japanese version validated.

### Subjective Socioeconomic Status (SES; childhood and current, seven items)

Assesses perceived SES during childhood (three items) and currently (four items). Items rated 1 to 6. Higher scores = greater perceived SES. Japanese version validated.

### Brief Parental Burnout Scale (BPBs, five items)

Assesses parental burnout behaviors. Items rated 0 to 2; total 0 to 10. Higher scores = greater burnout. Japanese version validated.

## Sleep Characteristics

### Insomnia Severity Index (ISI, seven items)

Assesses insomnia severity over the past two weeks. Items rated 0 to 4; total 0 to 28. Cut-offs: 0 to 7 (none), 8 to 14 (subthreshold), 15 to 21 (moderate), 22 to 28 (severe). Japanese version validated.

### Regularity, Satisfaction, Alertness, Timing, Efficiency, Duration of Sleep Scale (RU-SATED, six items)

Assesses multidimensional sleep health (regularity, satisfaction, alertness, timing, efficiency, duration). Each domain rated 0 to 2; total 0 to 12. Higher scores = greater sleep health. Japanese version validated.

### Reduced Morningness–Eveningness Questionnaire (rMEQ, five items)

Assesses chronotype. Total 4 to 25; lower scores = evening type, higher = greater morningness. Japanese version validated.

### Dysfunctional Beliefs and Attitudes about Sleep Scale (DBAS-16, 16 items)

Assesses maladaptive cognitions related to sleep. Items rated 0 to 10; higher scores = more dysfunctional beliefs. Japanese version validated.

### Ford Insomnia Response to Stress Test (FIRST, nine items)

Assesses vulnerability to stress-related insomnia. Items rated 1 to 4, total 9 to 36. Higher scores indicate greater vulnerability. Japanese version validated.

### Anxiety and Preoccupation about Sleep Questionnaire (APSQ, 10 items)

Assesses anxiety and preoccupation about sleep, including worries about sleep duration, health consequences, and loss of sleep control. Items rated on a Likert scale; higher scores indicate greater anxiety and preoccupation about sleep. Japanese version under validation.

### Bedtime Procrastination Scale (BPS, eight items)

Assesses tendency to delay bedtime. Items rated on a Likert scale, higher scores = greater procrastination. Japanese version validated, differing from the English original.

### Sleep Hygiene Practice Scale (SHPS, 30 items)

Assesses engagement in sleep-promoting behaviors. Items rated on a Likert scale, higher scores = better practices. Japanese version validated.

## Psychiatric Symptoms

### Patient Health Questionnaire-9 (PHQ-9, nine items)

Assesses depressive symptoms based on the DSM-IV criteria. Each item is rated on a four-point Likert scale (0=not at all to 3=nearly every day); total score 0 to 27. Higher scores indicate more severe depressive symptoms. A cut-off of ≥10 indicates probable major depressive disorder. Japanese version validated.

### Generalized Anxiety Disorder-7 (GAD-7, seven items)

Measures the core symptoms of generalized anxiety disorder. Items rated 0 to 3; total 0 to 21. Cut-offs: 5 (mild), 10 (moderate), 15 (severe). Japanese version validated.

### Liebowitz Social Anxiety Scale (LSAS, 24 items)

Assesses fear and avoidance in 24 social and performance situations. Each situation rated fear (0 to 3) and avoidance (0 to 3). Total 0 to 144. Higher scores indicate greater severity; ≥65 suggests marked social anxiety disorder. Japanese version validated.

### Anxiety Sensitivity Index-3 (ASI-3, 18 items)

Evaluates fear of anxiety-related sensations across the physical, cognitive, and social domains. Rated 0 to 4, total 0 to 72. Higher scores indicate greater anxiety sensitivity. Japanese version validated.

### Impact of Event Scale-Revised (IES-R, 22 items)

Assesses post-traumatic stress symptoms (intrusion, avoidance, hyperarousal). Items rated 0 to 4; total 0 to 88. Cut-off ≥33 indicates probable post-traumatic stress disorder. Japanese version validated.

### Obsessive/Compulsive Inventory-Revised (OCI-R, 18 items)

Measures severity of obsessive/compulsive symptoms. Items rated 0 to 4; total 0 to 72. Higher scores = more severe symptoms. Japanese version validated.

### Short Form of the Autism-Spectrum Quotient (AQ-28, 28 items)

Assesses autistic traits in adults across domains such as social skills, attention switching, attention to detail, communication, and imagination. Each item is rated on a four-point Likert scale and total scores range from 0 to 28, with higher scores indicating stronger autism spectrum traits. The AQ-28 is a short form of the original 50-item AQ. Japanese version validated.

### Adult ADHD Self-Report Scale (ASRS, 18 items)

Assesses symptoms of attention-deficit/hyperactivity disorder (ADHD) in adults based on the DSM-IV criteria. The scale comprises 18 items (Part A: six items; Part B: 12 items), each rated on a five-point Likert scale reflecting symptom frequency over the past six months. Higher scores indicate greater ADHD symptom severity. The ASRS is widely used as a screening instrument for adult ADHD. Japanese version validated.

## Attitudes toward Digital Health Technology

### eHealth Literacy Scale (eHEALS, eight items)

Assesses perceived skills in finding, evaluating, and applying online health information. Items rated 1 to 5; total 8 to 40. Higher = better literacy. Japanese version validated.

### Japanese Version of the e-Therapy Attitudes and Process Questionnaire (eTAP-J, 16 items)

Assesses intention, attitudes, norms, and control related to e-therapy. Items rated 1 to 7. Higher scores = stronger positive attitudes. To be validated in this study.

### Attitudes toward Safety, Support, Understanding, and Risk for Human-AI therapists (ASSURE-HT, 15 items; ASSURE-AI, 15 items)

Assesses attitudes toward human therapists (ASSURE-HT) and AI therapists (ASSURE-AI) providing psychotherapy. Items rated 1 to 5. Under the hypothesized two-factor model, higher ASSURE-HT inhibition scores indicate greater concerns about human therapists delivering psychotherapy (e.g., safety and trust concerns), whereas higher acceptance scores indicate more favorable attitudes toward human therapists (e.g., expected support and perceived benefit). The ASSURE-AI score reflects the perceived risk of psychotherapy by AI therapists from five perspectives (to be verified in this study).

### AI romance ethics (nine items)

Assesses ethical concerns about romantic relationships with AI. Items rated 1 to 7. Higher scores = more ethical concern.

### Anthropomorphism (18 items)

Assesses perceptions of AI agency and experience. Items rated 1 to 7. Higher = greater anthropomorphism.

### Fulfilling emotional needs (8 items)

Assesses the extent to which an interaction or relationship fulfils specific emotional needs (e.g., feeling less lonely, receiving comfort). Items are rated on a seven-point scale; the mean score reflects overall fulfillment (higher = greater fulfilment).

### Relationship Authenticity (RA, six items)

Assesses perceived intimacy/realness of AI relationships. Items rated 1 to 7. Higher = stronger authenticity.

### Desire for Real-World Relationships (DRR, six items)

Assesses desire to translate AI relationships into real-world human partnerships. Items rated 1 to 7. Higher = stronger desire.

**Supplementary Table 1. Overview of the variables assessed for the Transdiagnostic Assessment of Mental and Sleep (TAMS) registry**

| **Domain** | **Measure** | **Items and response scale** | **Score range / interpretation** |
| --- | --- | --- | --- |
| **QOL and psychosocial factors** | Japanese version of the Distress Questionnaire (DQ5-J) | 5 items; 5-point Likert | Higher = greater distress |
|  | Kessler Psychological Distress Scale (K6) | 6 items; 5-point Likert | Higher = greater psychological distress |
|  | EuroQol 5 Dimensions 5-Level (EQ-5D-5L) | 5 domains × 5 levels / visual analog scale | Higher = greater health-related QOL |
|  | Satisfaction With Life Scale (SWLS) | 5 items; 7-point Likert | Higher = greater life satisfaction |
|  | Short form of the Metacognitions Questionnaire (MCQ-30; positive beliefs about worry and the negative beliefs about uncontrollability and danger of worry subscales) | 12 items; 4-point Likert | Higher = greater maladaptive beliefs about worry |
|  | Effortful Control Scale (EC; attention control subscale) | 12 items; 4-point Likert | Sum or mean; higher = greater attentional control |
|  | Cognitive Attentional Syndrome Scale-1 Revised (CAS-1R; coping strategies subscale) | 6 items; 11-point Likert | Higher = greater use of coping strategies |
|  | Shift-and-Persist coping strategy (SAP) | 14 items; 6-point Likert | Higher = greater adaptive coping |
|  | Subjective socioeconomic status (SES; childhood and current) | 7 items; 6-point Likert | Higher = higher perceived SES |
|  | Brief Parental Burnout Scale (BPBs) | 5 items; 3-point Likert | Higher = greater burnout |
| **Sleep Characteristics** | Insomnia Severity Index (ISI) | 7 items; 5-point Likert | Higher = greater insomnia severity |
|  | Regularity, Satisfaction, Alertness, Timing, Efficiency, Duration of Sleep Scale (RU-SATED) | 6 items; 3-point Likert | Higher = greater sleep health |
|  | Reduced Morningness–Eveningness Questionnaire (rMEQ) | 5 items; 5-point Likert etc | Higher = greater morningness |
|  | Dysfunctional Beliefs and Attitudes about Sleep Scale (DBAS-16) | 16 items; 11-point Likert | Higher = greater dysfunctional beliefs |
|  | Ford Insomnia Response to Stress Test (FIRST) | 9 items; 4-point Likert | Higher = greater vulnerability to insomnia |
|  | Anxiety and Preoccupation about Sleep Questionnaire (APSQ) | 10 items; 10-point Likert | Higher scores = greater anxiety and preoccupation about sleep |
|  | Bedtime Procrastination Scale (BPS) | 8 items; 5-point Likert | Higher = greater bedtime procrastination |
|  | Sleep Hygiene Practice Scale (SHPS) | 30 items; 6-point Likert | Higher = better sleep hygiene |
| **Psychiatric symptoms** | Patient Health Questionnaire-9 (PHQ-9) | 9 items; 4-point Likert | Higher = greater depressive symptom severity |
|  | Generalized Anxiety Disorder-7 (GAD-7) | 7 items; 4-point Likert | Higher = greater anxiety symptom severity |
|  | Liebowitz Social Anxiety Scale (LSAS) | 24 items; 4-point Likert | Higher = greater social anxiety severity |
|  | Anxiety Sensitivity Index-3 (ASI-3) | 18 items; 5-point Likert | Higher = greater sensitivity |
|  | Impact of Event Scale-Revised (IES-R) | 22 items; 5-point Likert | Higher = greater post-traumatic stress symptom severity |
|  | Obsessive/Compulsive Inventory-Revised (OCI-R) | 18 items; 5-point Likert | Higher = greater obsessive/compulsive symptom severity |
|  | Short form of the Autism-Spectrum Quotient (AQ-28) | 28 items; 4-point Likert | Higher = greater autism spectrum traits |
|  | Adult ADHD Self-Report Scale (ASRS) | 18 items; 5-point Likert | Higher = greater ADHD symptom severity |
| **Attitudes toward digital health technology** | eHealth Literacy Scale (eHEALS) | 8 items; 5-point Likert | Higher = greater perceived eHealth literacy |
|  | Japanese version of the e-Therapy Attitudes and Process Questionnaire (eTAP-J) | 16 items; 7-point Likert | Higher = more positive attitudes |
|  | Attitudes toward Safety, Support, Understanding, and Risk for Human-AI therapists (ASSURE; two parallel scales: (ASSURE-HT and ASSURE-AI) | ASSURE-HT: 15 items  ASSURE-AI: 15 items  5-point Likert scales. | ASSURE-HT  Higher = greater (concerns/ favorable) of psychotherapy by human  ASSURE-AI  Higher = greater (concerns/ favorable) of psychotherapy by AI |
|  | AI romance ethics | 9 items; 7-point Likert | Higher = higher ethical concerns |
|  | Anthropomorphism | 18 items; 7-point Likert | Higher = greater anthropomorphism |
|  | Relationship Authenticity (RA) | 6 items; 7-point Likert | Higher = greater perceived authenticity |
|  | Desire for Real-World Relationships (DRR) | 6 items; 7-point Likert | Higher = greater desire for real-world relationships |
|  | Fulfilling emotional needs | 8 items; 7-point Likert | Higher = greater fulfillment of emotional needs |

# Details of the Analyses

## General Conventions

All the statistical tests will be two-sided. Multiple comparison corrections will be applied selectively as described within each sub-study. Model assumptions, including normality, homoscedasticity, and linearity, will be evaluated; if any are violated, appropriate transformations or non-parametric alternatives will be used. The potential non-representativeness of the study sample will also be considered. When appropriate, methods to adjust for sample bias will be applied, including weighting by demographic or clinical characteristics and propensity score-based adjustments.

## Aim 1: Psychosocial Correlations and Measurement Foundations for QOL and Psychiatric Symptoms

### Sub-study 1a. Reliability and Validity of the DQ5-J

In this study, we will validate the DQ5-J in patients with mental disorders, hypothesizing adequate internal consistency and two-week test–retest reliability, a unidimensional structure with a good model fit, expected convergent/discriminant/known-groups validity, and useful diagnostic accuracy. All the analyses will be conducted following the COSMIN guidelines [1].

Internal consistency will be evaluated using Cronbach’s α and McDonald’s ω. Test–retest reliability will be assessed using intraclass correlation coefficients in a subsample of participants who report “no change” on the PGIC at the two-week retest.

Structural validity will be examined using confirmatory factor analysis (CFA) to test the a priori unidimensional structure, with the model fit evaluated according to the following criteria: CFI and TLI ≥ 0.95, RMSEA ≤ 0.06, and SRMR ≤ 0.08. Exploratory factor analysis (EFA) will be conducted as a follow-up only if the model fit is inadequate.

Convergent validity will be assessed through the correlations with established measures of psychological distress and psychiatric symptoms (K6, PHQ-9, GAD-7). Based on prior validation studies, we hypothesize moderate-to-strong positive correlations (*r* ≥ 0.60). Discriminant validity will be evaluated by examining weak correlations (|*r*| < 0.30) with theoretically distinct constructs (eHEALS). Data distributions will be assessed using the Shapiro–Wilk test and visual inspection, and Pearson or Spearman correlation coefficients will be applied as appropriate.

Known-groups validity will be examined by comparing the DQ5-J scores between participants with mental disorders and those of a non-clinical control group using independent-samples t tests (or Welch’s t test or the Mann–Whitney U test if assumptions are violated). Large effect sizes (Cohen’s *d* ≥ 0.80) are hypothesized, consistent with prior validation studies.

Criterion validity will be evaluated using receiver operating characteristic analysis, with area under the curve and data-driven cutoff values used to assess diagnostic utility for detecting mental disorders.

### Sub-study 1b. Parental Burnout Behaviors in Relation to Socioeconomic Status, Mental Health, and Sleep

In this study, we will examine the associations between the BPBs scores and a comprehensive set of correlates spanning multiple psychosocial domains: mental health (depressive symptoms [PHQ-9], anxiety symptoms [GAD-7], and general psychological distress [K6]), sleep-related outcomes (insomnia severity [ISI] and multidimensional sleep health [RU-SATED]), subjective well-being and health-related QOL (life satisfaction [SWLS] and EQ-5D-5L), individual-difference and cognitive-regulatory factors (effortful control [EC], CAS coping strategies [CAS-1R], and maladaptive metacognitive beliefs [MCQ-30]), current SES, and neurodevelopmental traits (ADHD traits [ASRS] and autistic traits [AQ-28]).

Before the analyses, the distributional properties of all the variables (normality, skewness, and kurtosis) will be examined. Pearson’s correlation coefficients will be used for approximately normally distributed variables, whereas Spearman’s rank correlations (*ρ*) will be applied when substantial deviations from normality are observed. Outliers will be identified using standardized residuals (|*z*| > 3.0) and sensitivity analyses excluding these observations will be reported.

We hypothesize that higher scores will be associated with greater depressive and anxiety symptoms and general psychological distress, poorer sleep quality, lower life satisfaction and health-related QOL, weaker effortful control, stronger maladaptive cognitive attentional and metacognitive tendencies, lower subjective SES, and higher ADHD and ASD traits. Correlation analyses, including partial correlations controlling for age, sex, and other prespecified demographic variables, will be conducted. To address multiple testing, the false discovery rate (FDR) will be controlled for using the Benjamini–Hochberg procedure (*q* < .05) within each prespecified conceptual family (mental health, sleep, well-being/QOL, individual differences, SES, and neurodevelopmental traits). The outcomes within each family will be treated as a single multiplicity set given their expected correlations.

### Sub-study 1c. Shift-and-persist Coping Strategies in Relation to Mental Disorders

In this study, we will examine whether individual differences in shift-and-persist coping strategies are associated with case–control status across multiple mental disorders. Using a panel-based case–control design, each diagnostic group—comprising patients with major depressive disorder, social anxiety disorder, panic disorder, post-traumatic stress disorder, and obsessive/compulsive disorder—will be compared separately with a shared non-clinical control group. Consistent with prior work [2], we hypothesize that higher SAP scores will be associated with a lower likelihood of belonging to patient groups relative to healthy controls across all the examined disorders.

SAP will be treated as the primary exposure and categorized into quartiles for the main analyses. Descriptive comparisons across these quartiles will be conducted using chi-square tests and ordinal trends in case–control status across SAP quartiles will be assessed using Cochran–Armitage trend tests. These trend tests are conducted as descriptive supplementary analyses to characterize the shape of the association across the SAP distribution; they are not subject to FDR correction and inferential emphasis is placed on the logistic regression models described below.

For each mental disorder, logistic regression models will be fitted to estimate odds ratios and 95% confidence intervals, adjusting for prespecified covariates, including age, sex, education, and SES (childhood and current). For diagnostic groups in which the events per variable ratio falls below 10, Firth-penalized logistic regression will be used to improve the estimation stability and small-sample bias [3,4]. Multiple testing across disorders will be addressed using the FDR. Given the multiple diagnostic groups examined, the FDR will be controlled for using the Benjamini–Hochberg procedure. Statistical significance will be evaluated using an FDR-adjusted q-value threshold of 0.05. Sensitivity analyses will be conducted by modeling SAP as a continuous variable.

### Sub-study 1d. Metacognitive Beliefs, CAS, and Attentional Control in Relation to QOL and Psychiatric Symptoms

This sub-study will examine the transdiagnostic and diagnosis-specific associations and conditional pathways linking metacognitive beliefs, attentional control, QOL, and diagnosis-specific symptoms, with a focus on CAS, across patients with major depressive disorder, social anxiety disorder, post-traumatic stress disorder, obsessive/compulsive disorder, and panic disorder as well as the non-clinical control group.

**Analytic overview**

The analytic strategy comprises three complementary components that address the (i) variable-centered pathway models, (ii) person-centered (profile-based) heterogeneity in CAS coping strategies, and (iii) supplementary profiling of metacognitive beliefs and attentional control to aid the interpretation of the primary models. All the analyses will be cross-sectional and hypothesis-driven unless otherwise specified.

**Measures and variables**

Metacognitive beliefs will be assessed using two MCQ-30 subscales: positive beliefs about worry and negative beliefs about uncontrollability and danger of worry. These subscales will be examined either jointly or separately depending on the model specification. CAS will be measured using the six-item coping strategies subscale of the CAS-1R, operationalized as a composite (sum) score in all the variable-centered models. Attentional control will be assessed using the attentional control subscale of the EC. QOL will be measured using the EQ-5D-5L; this will serve as the primary outcome in all the groups. Diagnosis-specific symptom outcomes will be included as secondary outcomes: the PHQ-9 for major depressive disorder, the LSAS for social anxiety disorder, the IES-R for post-traumatic stress disorder, the OCI-R for obsessive/compulsive disorder, and the ASI-3 for panic disorder (exploratory). Symptom outcomes will not be used as primary outcomes in the non-clinical control group.

Primary analyses will use the total scores for each symptom measure. For instruments with established subscales (LSAS, IES-R, OCI-R, ASI-3), exploratory or sensitivity analyses may additionally be used to examine subscale-level outcomes to evaluate dimensional specificity.

**Multiple-group structural equation modeling (SEM) for moderated mediation**

Multiple-group SEM will be used to estimate moderated mediation models, comparing each clinical group separately with the non-clinical control. In each model, metacognitive beliefs predict QOL indirectly via CAS, with attentional control specified as a moderator of the CAS-to-QOL path (i.e., a conditional indirect effect framework). Attentional control will also be tested as a moderator of the metacognitive beliefs-to-CAS path and CAS-to-symptom path in the symptom models, consistent with the primary aim of moderated mediation. Model specification and interpretation will follow standard guidance for moderated mediation [5]. The direct effects of metacognitive beliefs on QOL will also be estimated.

Group differences will be evaluated by constraining and freeing the corresponding path coefficients across the groups and comparing the model fit using likelihood ratio tests and changes in fit indices. Given that symptom measures show quantitative continuity across clinical and non-clinical populations, all the outcomes will be treated as continuous; however, distributions (including floor effects, variance, and skewness) will be examined within each group before modeling. If pronounced floor effects are detected for a given symptom measure, the group-comparison results for that outcome will be interpreted cautiously and within-disorder models will be reported in sensitivity analyses.

**Latent profile analysis of CAS coping strategies**

To examine the heterogeneity in CAS-related coping patterns, latent profile analysis will be conducted on the six-item coping strategies subscales of the CAS-1R. Profiles will be estimated using maximum likelihood with robust standard errors. Model selection will be based on the Bayesian information criterion, entropy, class interpretability, and theoretical plausibility.

After class enumeration, profile membership and class proportions will be summarized within each diagnostic group and the differences across the groups will be described. Associations between the CAS profiles and outcomes (EQ-5D-5L and symptom severity) will be tested using the Bolck–Croon–Hagenaars method for continuous outcomes and the three-step approach for categorical/distal outcomes (three-step approach for distal outcomes in latent class/profile analysis/related three-step implementations), as appropriate, accounting for classification uncertainty [6,7].

Where a binary EQ-5D-5L outcome is additionally examined (exploratory), deterioration will be defined using a minimal important difference-based threshold; this will be considered a supplementary specification rather than a required primary endpoint.

**Supplementary latent profile analysis of metacognitive beliefs and attentional control**

As a supplementary analysis, latent profile analysis will be conducted on metacognitive beliefs (two subscales of the MCQ-30) and attentional control (attentional control subscale of the EC). This analysis will aim to clarify whether distinct configurations of metacognitive beliefs and attentional control correspond to the CAS profiles and QOL outcomes, supporting the interpretation of the moderated mediation models. The results of this analysis will be considered exploratory and reported separately from the primary hypotheses. Person-centered profiling will complement, rather than replace, the variable-centered models [8].

**Statistical considerations**

Two-sided tests will be used throughout. For SEM, robust standard errors will be used where appropriate; model convergence and improper solutions will be checked (e.g., non-positive definite matrices, inadmissible parameter estimates), and alternative optimizers or starting values will be applied as needed. Effect sizes with 95% confidence intervals will be reported for all the key parameters. Sensitivity analyses will include repeating the moderated mediation models within each clinical group (excluding the non-clinical control group) for those symptom outcomes for which marked floor effects are observed in the non-clinical control group.

Multiplicity will be addressed by analytic family. For the primary CFAs (i.e., the multiple-group SEM models evaluating QOL as the primary outcome across each clinical group compared with the non-clinical control group), the family-wise error will be controlled for using the Holm procedure across the prespecified primary hypothesis tests, where applicable. For the secondary analyses and EFAs, including the diagnosis-specific symptom outcomes, subscale-level sensitivity analyses, profile–outcome associations in latent profile analysis, and supplementary profiling analyses, the FDR will be controlled for using the Benjamini–Hochberg procedure within each analytic family, where appropriate. The model selection indices for latent profile enumeration (e.g., Bayesian information criterion, entropy, and interpretability) and descriptive statistics will not be multiplicity-adjusted, as they are not interpreted as formal hypothesis tests. Because several outcomes are expected to be correlated, the FDR control will be applied within families of related outcomes rather than across all the tests combined. Multiplicity adjustments will be implemented in R based on the *p*-values obtained from Mplus outputs, when applicable.

## Aim 2: Sleep Characteristics and Psychiatric Symptom Profiles

### Sub-study 2a. Sleep Profiles in Patients with Mental Disorders

In this study, we will conduct data-driven phenotyping based on sleep-related indicators in patients with mental disorders. First, we will identify diagnosis-transcending sleep phenotypes across all psychiatric disorders. Next, if sufficient sample sizes are obtained for each disorder and the non-clinical control group, we will identify disease-specific sleep phenotypes.

Age is a potential confounder, as it strongly influences sleep-related measures. Accordingly, the sleep-related variables used for clustering will be adjusted for age using residualization based on linear regression models, with age entered as an explanatory variable [9]. The resulting age-adjusted residuals will be used in all the subsequent clustering analyses.

Dimensionality reduction will first be performed using sleep-related variables, including ISI, RU-SATED, rMEQ, DBAS, FIRST, APSQ, BPS, SHPS, subjective sleep duration, social jetlag, sleep midpoint, number of nap days, and nap duration. Uniform manifold approximation and projection (UMAP) will be applied to visualize the latent structure of these variables in a low-dimensional space [10].

Based on the distribution and apparent cluster structure observed in the UMAP embeddings, multiple clustering methods, including Density-Based Spatial Clustering of Applications with Noise, hierarchical clustering, k-means, and Gaussian mixture models, will be applied. The clustering method most appropriate for the data structure will be selected and the phenotype will be classified accordingly. The optimal number of clusters will be determined in accordance with the comprehensive framework for clustering validation described by Hassan et al. [11], integrating multiple internal validation criteria. When these indices suggest different optimal cluster numbers, solutions consistently supported across multiple indices will be prioritized.

To evaluate cluster stability, resampling-based stability analyses will be conducted. Specifically, subsampling procedures will be applied and consensus clustering will be used to assess the consistency of cluster assignments. Agreement between the clustering solutions obtained by reapplying UMAP and the selected clustering method will be quantified using the adjusted Rand index and Jaccard index.

Differences between the identified clusters will be examined using psychological and demographic variables. For continuous variables, normality will be assessed; one-way analysis of variance (ANOVA) followed by Tukey’s HSD post-hoc test will be applied when the assumptions are met, while the Kruskal–Wallis test followed by Steel–Dwass post-hoc tests will be used otherwise. Categorical variables will be compared using chi-square tests. Given the exploratory nature of these cluster comparisons, no additional correction across the outcome variables will be applied beyond the post-hoc tests specified above.

### Sub-study 2b. Sleep Profiles in Patients with Diabetes

In this study, we will conduct data-driven phenotyping based on sleep-related indicators in patients with type 1 and type 2 diabetes. First, we will identify diagnosis-transcending sleep phenotypes among the patients.
Next, we will explore sleep phenotypes specific to type 1 and type 2 diabetes and the non-clinical control group, respectively.

Again, age is a potential confounder, as it strongly influences sleep-related measures. Moreover, age distributions are expected to differ across type 1 and type 2 diabetes because of disease-specific characteristics. To account for this, the sleep-related variables used for clustering will be adjusted for age using residualization based on linear regression models, with age entered as an explanatory variable [9]. The resulting age-adjusted residuals will be used in all the subsequent clustering analyses.

The clustering procedures, determination of the optimal number of clusters, and stability analyses will be conducted using the same methods as described in Sub-study 2a. Differences between the identified clusters will be examined using psychological and demographic variables. For continuous variables, normality will be assessed; one-way ANOVA followed by Tukey’s HSD post-hoc test will be applied when the assumptions are met, while the Kruskal–Wallis test followed by Steel–Dwass post-hoc tests will be used otherwise. Categorical variables will be compared using chi-square tests. Given the exploratory nature of these cluster comparisons, no additional correction across the outcome variables will be applied beyond the post-hoc tests specified above.

### Sub-study 2c. Psychiatric profiles in Patients with Developmental Disabilities

In this study, we will conduct a data-driven phenotype analysis based on the psychiatric symptoms of individuals with developmental disabilities (ASD, ADHD). First, we will identify cross-diagnostic psychiatric symptom phenotypes for ASD and ADHD. Next, if sufficient sample sizes are obtained for each disorder, we will identify psychiatric symptom phenotypes specific to each disability. Dimensionality reduction will first be performed using the psychiatric symptom variables, including PHQ-9, GAD-7, LSAS, ASI-3, IES-R, OCI-R, and K6. UMAP will be applied to visualize the latent structure of these variables in a low-dimensional space [10].

The clustering procedures, determination of the optimal number of clusters, and stability analyses will be conducted using the same methods as described in Sub-study 2a. Differences between identified clusters will be examined using developmental trait measures (AQ-28 and ASRS) as well as the SWLS, the EQ-5D-5L, and demographic variables. For continuous variables, normality will be assessed; one-way ANOVA followed by Tukey’s HSD post-hoc test will be applied when the assumptions are met, while the Kruskal–Wallis test followed by Steel–Dwass post-hoc tests will be used otherwise. Categorical variables will be compared using chi-square tests. Given the exploratory nature of these cluster comparisons, no additional correction across the outcome variables will be applied beyond the post-hoc tests specified above.

## Aim 3: Attitudes toward Digital Health Technology

### Sub-study 3a. Reliability and Validity of the Japanese Version of e-Therapy Attitudes and Process Questionnaire (eTAP-J)

In this study, we will examine the validity of the eTAP-J in healthy participants and patients with mental disorders. We hypothesize that the scale will exhibit adequate internal consistency; a four-factor structure with a satisfactory model fit; and evidence of convergent, discriminant, and known-groups validity.

Internal consistency will be evaluated using Cronbach’s *α* and McDonald’s *ω*, while the factor structure will be examined using CFA. The model fit will be considered acceptable if the RMSEA and SRMR values are ≤ 0.08 and the CFI and TLI values are ≥ 0.90. More stringent criteria for a good model fit will be defined as CFI and TLI ≥ 0.95 and RMSEA ≤ 0.06. If the CFA does not yield an acceptable model fit, EFA will be conducted.

Convergent and discriminant validity will be assessed using correlation analyses. We hypothesize that the total score of the eTAP-J (or the Perceived Behavioral Control factor score) will show a moderately strong positive correlation with the total score of the eHEALS (*r* = 0.30–0.50). By contrast, the eTAP-J scores are expected to show a weak correlation with the total score of the SWLS (|*r*| < 0.30), indicating adequate discriminant validity.

Known-groups validity will be examined by comparing the total eTAP-J scores using t-tests. We hypothesize that participants with a history of psychological support or treatment will score significantly higher on the eTAP-J than those without such experience (Cohen’s *d* ≥ 0.50) and that patients with mental disorders will score significantly higher than the non-clinical control group (Cohen’s *d* ≥ 0.50). However, if only configural invariance is supported in the factor analysis, no group comparisons between patients with mental disorders and the non-clinical control group will be conducted.

The primary analyses for convergent validity, discriminant validity, and known-groups validity are based on a priori hypotheses and a limited number of planned comparisons; therefore, no correction for multiple comparisons will be applied to these tests. By contrast, exploratory or post-hoc analyses involving multiple comparisons, such as analyses of the subscale scores and additional associations, will be adjusted using the Benjamini–Hochberg FDR procedure.

### Sub-study 3b. Reliability and Validity of ASSURE

In this study, we will develop two matched self-report 15-item scales assessing attitudes toward the use of human and AI therapists in terms of safety, support, understanding, and risk, termed ASSURE-HT and ASSURE-AI, respectively. Each scale will be treated as an independent measure in the analyses.

Based on prior theoretical and empirical work, the initial measurement model will be prespecified as a two-factor structure reflecting (a) expected support/understanding and perceived benefit and (b) perceived risk and safety concerns.

Psychometric evaluation will be conducted sequentially. All the validation samples will consist of individuals registered as panel monitors with diagnosed mental disorders, including major depressive disorder, social anxiety disorder, panic disorder, post-traumatic stress disorder, obsessive/compulsive disorder, insomnia, attention-deficit/hyperactivity disorder, and autism spectrum disorder.

First, structural validity will be examined using a CFA of the prespecified two-factor model, conducted separately for ASSURE-HT and ASSURE-AI in an initial sample (Sample 1). The model fit will be evaluated using the CFI, TLI, RMSEA, and SRMR, with an acceptable fit defined as CFI and TLI ≥ 0.95, RMSEA ≤ 0.06, and SRMR ≤ 0.08. The CFA sample size will be set at a minimum of 10 participants per item. If the prespecified model does not meet these criteria for either scale, EFA will be conducted in Sample 1 to refine the measurement model, followed by the CFA of the revised structure in an independent validation sample (Sample 2).

Second, internal consistency reliability will be examined for each factor and for the total score (where appropriate) separately for ASSURE-HT and ASSURE-AI using Cronbach’s *α* and McDonald’s *ω* (acceptable threshold: ≥ 0.70).

Third, measurement invariance and form comparability will be evaluated to support the treatment of ASSURE-HT and ASSURE-AI as matched forms for parallel comparisons. Measurement invariance will be tested using multi-group CFA across both scales and, where applicable, across the key demographic subgroups (e.g., sex and age groups), using pooled data from Samples 1 and 2. Configural and metric invariance will be assessed, with scalar invariance additionally examined if mean comparisons are planned. Subgroup sizes will be balanced as much as possible to facilitate interpretability. Invariance will be evaluated using changes in the fit indices, with *Δ*CFI ≤ 0.010 and *Δ*RMSEA ≤ 0.015 (and/or *Δ*SRMR ≤ 0.010 for metric invariance and ≤ 0.015 for scalar invariance) indicating support for invariance. If full invariance is not achieved, partial invariance will be explored by freeing non-invariant parameters, and cross-form or between-group comparisons will be limited accordingly.

Following the establishment of an acceptable measurement model and adequate reliability, construct validity will be examined through hypothesis testing. Convergent and discriminant validity will be examined using the eTAP-J, with higher scores indicating more positive attitudes toward e-therapy; weak-to-moderate correlations with the ASSURE scores are expected. Known-groups validity will be evaluated by examining differences in the ASSURE scores by prior face-to-face psychotherapy experience. Criterion validity will not be assessed because of the absence of a gold standard for psychotherapy risk perception. Responsiveness will also not be evaluated, as the registry does not include interventions intended to change attitudes.

### Sub-study 3c. Ethical Concerns about Romantic Relationships with AI

In this study, we will examine the extent to which patients with mental health conditions consider ethical concerns about romantic relationships with AI. We hypothesize that lower relationship satisfaction will be associated with stronger positive and negative perceptions of romantic relationships with AI and greater ethical concerns. Additionally, we hypothesize that higher perceived anthropomorphism will predict stronger relationship authenticity, which in turn will predict a greater desire for real-world AI relationships and higher fulfillment of emotional needs. Correlation analyses will be conducted among relationship satisfaction, AI romance ethics, relationship authenticity, desire for real-world AI relationships, and fulfillment of emotional needs. A serial mediation model will be tested using the PROCESS macro, with anthropomorphism as the predictor, relationship authenticity and desire for real-world AI relationships as the first and second mediators, and fulfillment of emotional needs as the outcome.

# Data Governance

This section describes the data governance for the online registry survey dataset used for the cross-sectional studies and two-week retest subsample. No formal Patient and Public Involvement (PPI) statement was made for registry design or governance.

## Capture and Identifiers

Participant recruitment, invitations, and two-week retest contact will be managed by the survey vendor (Macromill, Inc.) according to standard procedures. At the beginning of the survey, participants will be presented with a pledge item asking them to commit to responding carefully and only those who agree will be allowed to proceed to the subsequent questions [12]. Only complete, anonymized cases with project-specific study IDs will be delivered to the research team; incomplete responses will be excluded prior to delivery. The research team will not receive direct identifiers and will not hold mapping between study IDs and panel identities. The vendor will retain its own recontact key solely for retest invitations.

## Data Management and Sharing in Accordance with the FAIR Principles [13]

### Findable

All registry datasets and associated metadata will be assigned a DataCite DOI to ensure persistent and unique identification. The registry governing body will be responsible for DOI registration, maintenance, and long-term preservation together with the associated metadata. Publicly available metadata will include essential descriptive information such as the number of participants, instrument names, variable lists, data collection periods, and policies for handling missing data. Metadata records will explicitly reference the dataset DOI and specify access conditions and licensing information, ensuring a clear link between metadata and data objects. Metadata will be deposited in Zenodo in multiple machine-readable formats, enabling indexing by general search engines and scholarly data discovery services.

### Accessible

Registry data and metadata will be deposited in a trusted data repository with an assigned DOI and accessible through secure HTTPS-based communication. As the online survey dataset will contain no direct personal identifiers, the data will be released as open data, directly downloadable from the DOI landing page without a formal access request. Conditions for data use and reuse will be clearly specified under a CC BY 4.0 license. If access policies are modified in the future for ethical or legal reasons, the metadata will remain openly available via the DOI landing page, with clear information on data availability, access conditions, and contact details.

### Interoperable

Data and metadata will be structured using machine-readable standard formats and shared vocabularies. The dataset will include participant attributes, survey item responses, and derived summary scores organized systematically. The metadata and data dictionary will document instrument names, item identifiers, variable names, response scales, and definitions of derived scores using internationally recognized metadata standards and shared PROM-related vocabularies, supporting semantic consistency and interoperability within mental health research. To facilitate future linkage with clinical and healthcare datasets, compatibility with standards such as HL7 FHIR and, where appropriate, mapping to vocabularies such as LOINC will be considered.

### Reusable

Clear reuse conditions will be applied under a CC BY 4.0 license, permitting both non-commercial and commercial use. License terms and usage conditions will be documented on the dataset DOI landing page. Data provenance will be maintained, including survey administration procedures, recruitment methods, response modes, instrument versions, and scoring algorithms. Data processing steps such as ingestion, cleaning, recoding, variable derivation, and versioning will be recorded in a structured provenance framework to ensure transparency, auditability, and reproducibility. Semantic information, including instrument names, item identifiers, variable definitions, constructs, and derived scores, will be systematically organized in a data dictionary. Data quality assurance procedures, including range checks, internal consistency checks, and strict version control, will be applied to support long-term reuse.

**6. Checklists**

**Supplementary Table 2.** STROBE Statement—checklist of items that should be included in reports of observational studies

|  | **Item No.** | **Recommendation** | **Line No.** | **Relevant text from manuscript** |
| --- | --- | --- | --- | --- |
| **Title and abstract** | 1 | (*a*) Indicate the study’s design with a commonly used term in the title or the abstract | 1–27 | Title and Abstract |
|  |  | (*b*) Provide in the abstract an informative and balanced summary of what was done and what was found | 3–27 | Abstract |
| **Introduction** | | | |  |
| Background  /rationale | 2 | Explain the scientific background and rationale for the investigation being reported | 32–89 | Introduction |
| Objectives | 3 | State specific objectives, including any prespecified hypotheses | 90–99 | Introduction |
| **Methods** | | | |  |
| Study design | 4 | Present key elements of study design early in the paper | 101–107 | Material and Methods – Design |
| Setting | 5 | Describe the setting, locations, and relevant dates, including periods of recruitment, exposure, follow-up, and data collection | 114–133 | Material and Methods – Participants |
| Participants | 6 | (*a*) *Cohort study*—Give the eligibility criteria, and the sources and methods of selection of participants. Describe methods of follow-up  *Case-control study*—Give the eligibility criteria, and the sources and methods of case ascertainment and control selection. Give the rationale for the choice of cases and controls  *Cross-sectional study*—Give the eligibility criteria, and the sources and methods of selection of participants | 114-133 | Material and Methods – Participants  Supplementary Materials |
|  |  | (*b*) *Cohort study*—For matched studies, give matching criteria and number of exposed and unexposed  *Case-control study*—For matched studies, give matching criteria and the number of controls per case | NA | NA |
| Variables | 7 | Clearly define all outcomes, exposures, predictors, potential confounders, and effect modifiers. Give diagnostic criteria, if applicable | NA | Table 1  Supplementary Materials |
| Data sources/ measurement | 8* | For each variable of interest, give sources of data and details of methods of assessment (measurement). Describe comparability of assessment methods if there is more than one group | 114–133 | Material and Methods – Participants |
| Bias | 9 | Describe any efforts to address potential sources of bias | 213–222 | Material and Methods – Statistical Analysis |
| Study size | 10 | Explain how the study size was arrived at | 196–211 | Material and Methods – Sample Size and Sampling |

| Quantitative variables | 11 | Explain how quantitative variables were handled in the analysis. If applicable, describe which groupings were chosen and why | 212–249 | Material and Methods – Statistical Analysis  Supplementary Materials |
| --- | --- | --- | --- | --- |
| Statistical methods | 12 | (*a*) Describe all statistical methods, including those used to control for confounding | 212–249 | Material and Methods – Statistical Analysis  Supplementary Materials |
|  |  | (*b*) Describe any methods used to examine subgroups and interactions | NA | Supplementary Materials |
|  |  | (*c*) Explain how missing data were addressed | NA | NA  (Because no missing data) |
|  |  | (*d*) *Cohort study*—If applicable, explain how loss to follow-up was addressed  *Case-control study*—If applicable, explain how matching of cases and controls was addressed  *Cross-sectional study*—If applicable, describe analytical methods taking account of sampling strategy | NA | NA |
|  |  | (*e*) Describe any sensitivity analyses | 212–249 | Material and Methods - Statistical Analysis  Supplementary Materials |
| **Results** | | | | |
| Participants | 13* | (a) Report numbers of individuals at each stage of study—e.g., numbers potentially eligible, examined for eligibility, confirmed eligible, included in the study, completing follow-up, and analyzed | NA | NA |
|  |  | (b) Give reasons for non-participation at each stage | NA | NA |
|  |  | (c) Consider use of a flow diagram | NA | NA |
| Descriptive data | 14* | (a) Give characteristics of study participants (e.g., demographic, clinical, social) and information on exposures and potential confounders | NA | NA |
|  |  | (b) Indicate number of participants with missing data for each variable of interest | NA | NA |
|  |  | (c) *Cohort study*—Summarize follow-up time (e.g., average and total amount) | NA | NA |
| Outcome data | 15* | *Cohort study*—Report numbers of outcome events or summary measures over time | NA | NA |
|  |  | *Case-control study—*Report numbers in each exposure category, or summary measures of exposure | NA | NA |
|  |  | *Cross-sectional study—*Report numbers of outcome events or summary measures | NA | NA |
| Main results | 16 | (*a*) Give unadjusted estimates and, if applicable, confounder-adjusted estimates and their precision (e.g., 95% confidence interval). Make clear which confounders were adjusted for and why they were included | NA | NA |
|  |  | (*b*) Report category boundaries when continuous variables were categorized | NA | NA |
|  |  | (*c*) If relevant, consider translating estimates of relative risk into absolute risk for a meaningful time period | NA | NA |

| Other analyses | 17 | Report other analyses done—e.g., analyses of subgroups and interactions, and sensitivity analyses | NA | NA |
| --- | --- | --- | --- | --- |
| **Discussion** | | | | |
| Key results | 18 | Summarize key results with reference to study objectives | 295–302 | Discussion |
| Limitations | 19 | Discuss limitations of the study, taking into account sources of potential bias or imprecision. Discuss both direction and magnitude of any potential bias | 325–361 | Discussion |
| Interpretation | 20 | Give a cautious overall interpretation of results considering objectives, limitations, multiplicity of analyses, results from similar studies, and other relevant evidence | NA | NA (This manuscript reports a study protocol; no results are presented) |
| Generalizability | 21 | Discuss the generalizability (external validity) of the study results | 325–332 | Discussion |
| **Other information** | |  | | |
| Funding | 22 | Give the source of funding and the role of the funders for the present study and, if applicable, for the original study on which the present article is based | 412–416 | Funding |

*Give information separately for cases and controls in case-control studies and, if applicable, for exposed and unexposed groups in cohort and cross-sectional studies.

**Note:** An Explanation and Elaboration article discusses each checklist item and gives methodological background and published examples of transparent reporting. The STROBE checklist is best used in conjunction with this article (freely available on the Web sites of PLoS Medicine at http://www.plosmedicine.org/, Annals of Internal Medicine at http://www.annals.org/, and Epidemiology at http://www.epidem.com/). Information on the STROBE Initiative is available at www.strobe-statement.org.

**Supplementary Table 3.** Checklist for Reporting Results of Internet E-Surveys (CHERRIES)

| ***Checklist Item*** | ***Explanation*** | ***Line Number*** |
| --- | --- | --- |
| Describe survey design | Describe target population, sample frame. Is the sample a convenience sample? (In “open” surveys this is most likely.) | 114–133, 195–216 |
| IRB approval | Mention whether the study has been approved by an IRB. | 276–294 |
| Informed consent | Describe the informed consent process. Where were the participants told the length of time of the survey, which data were stored and where and for how long, who the investigator was, and the purpose of the study? | 126–133 |
| Data protection | If any personal information was collected or stored, describe what mechanisms were used to protect unauthorized access. | NA |
| Development and testing | State how the survey was developed, including whether the usability and technical functionality of the electronic questionnaire had been tested before fielding the questionnaire. | None |
| Open survey versus closed survey | An “open survey” is a survey open for each visitor of a site, while a closed survey is only open to a sample which the investigator knows (password-protected survey). | 114–133 |
| Contact mode | Indicate whether or not the initial contact with the potential participants was made on the Internet. (Investigators may also send out questionnaires by mail and allow for Web-based data entry.) | 134–141 |
| Advertising the survey | How/where was the survey announced or advertised? Some examples are offline media (newspapers), or online (mailing lists – If yes, which ones?) or banner ads (Where were these banner ads posted and what did they look like?). It is important to know the wording of the announcement as it will heavily influence who chooses to participate. Ideally the survey announcement should be published as an appendix. | 114–125 |
| Web/E-mail | State the type of e-survey (e.g., one posted on a Web site, or one sent out through e-mail). If it is an e-mail survey, were the responses entered manually into a database, or was there an automatic method for capturing responses? | 114–125 |
| Context | Describe the Web site (for mailing list/newsgroup) in which the survey was posted. What is the Web site about, who is visiting it, what are visitors normally looking for? Discuss to what degree the content of the Web site could pre-select the sample or influence the results. For example, a survey about vaccination on an anti-immunization Web site will have different results from a Web survey conducted on a government Web site | 114–125 |
| Mandatory/voluntary | Was it a mandatory survey to be filled in by every visitor who wanted to enter the Web site, or was it a voluntary survey? | 134–141 |
| Incentives | Were any incentives offered (e.g., monetary, prizes, or non-monetary incentives such as an offer to provide the survey results)? | 134–141 |
| Time/Date | In what timeframe were the data collected? | 115–116 |
| Randomization of items or questionnaires | To prevent biases items can be randomized or alternated. | None |
| Adaptive questioning | Use adaptive questioning (certain items, or only conditionally displayed based on responses to other items) to reduce number and complexity of the questions. | NI |
| Number of Items | What was the number of questionnaire items per page? The number of items is an important factor for the completion rate. | 166-167 |
| Number of screens (pages) | Over how many pages was the questionnaire distributed? The number of items is an important factor for the completion rate. | 166-167 |
| Completeness check | It is technically possible to do consistency or completeness checks before the questionnaire is submitted. Was this done, and if “yes,” how (usually JavaScript)? An alternative is to check for completeness after the questionnaire has been submitted (and highlight mandatory items). If this has been done, it should be reported. All items should provide a non-response option such as “not applicable” or “rather not say,” and selection of one response option should be enforced. | NI |
| Review step | State whether respondents were able to review and change their answers (e.g., through a Back button or a Review step which displays a summary of the responses and asks the respondents if they are correct). | NI |
| Unique site visitor | If you provide view rates or participation rates, you need to define how you determined a unique visitor. There are different techniques available, based on IP addresses or cookies or both. | NI |
| View rate (Ratio of unique survey visitors/unique site visitors) | Requires counting unique visitors to the first page of the survey, divided by the number of unique site visitors (not page views!). It is not unusual to have view rates of less than 0.1 % if the survey is voluntary. | NI |
| Participation rate (Ratio of unique visitors who agreed to participate/unique first survey page visitors) | Count the unique number of people who filled in the first survey page (or agreed to participate, for example by checking a checkbox), divided by visitors who visit the first page of the survey (or the informed consents page, if present). This can also be called “recruitment” rate. | NI |
| Completion rate (Ratio of users who finished the survey/users who agreed to participate) | The number of people submitting the last questionnaire page, divided by the number of people who agreed to participate (or submitted the first survey page). This is only relevant if there is a separate “informed consent” page or if the survey goes over several pages. This is a measure for attrition. Note that “completion” can involve leaving questionnaire items blank. This is not a measure for how completely questionnaires were filled in. (If you need a measure for this, use the word “completeness rate.”) | NI |
| Cookies used | Indicate whether cookies were used to assign a unique user identifier to each client computer. If so, mention the page on which the cookie was set and read, and how long the cookie was valid. Were duplicate entries avoided by preventing users access to the survey twice; or were duplicate database entries having the same user ID eliminated before analysis? In the latter case, which entries were kept for analysis (e.g., the first entry or the most recent)? | 251–263 |
| IP check | Indicate whether the IP address of the client computer was used to identify potential duplicate entries from the same user. If so, mention the period of time for which no two entries from the same IP address were allowed (e.g., 24 hours). Were duplicate entries avoided by preventing users with the same IP address access to the survey twice; or were duplicate database entries having the same IP address within a given period of time eliminated before analysis? If the latter, which entries were kept for analysis (e.g., the first entry or the most recent)? | NI |
| Log file analysis | Indicate whether other techniques to analyze the log file for identification of multiple entries were used. If so, please describe. | NI |
| Registration | In “closed” (non-open) surveys, users need to login first and it is easier to prevent duplicate entries from the same user. Describe how this was done. For example, was the survey never displayed a second time once the user had filled it in, or was the username stored together with the survey results and later eliminated? If the latter, which entries were kept for analysis (e.g., the first entry or the most recent)? | 281–283 |
| Handling of incomplete questionnaires | Were only completed questionnaires analyzed? Were questionnaires which terminated early (where, for example, users did not go through all questionnaire pages) also analyzed? | NA |
| Questionnaires submitted with an atypical timestamp | Some investigators may measure the time people needed to fill in a questionnaire and exclude questionnaires that were submitted too soon. Specify the timeframe that was used as a cut-off point, and describe how this point was determined. | NA |
| Statistical correction | Indicate whether any methods such as weighting of items or propensity scores have been used to adjust for the non-representative sample; if so, please describe the methods. | Supplementary Materials |

This checklist has been modified from Eysenbach G. Improving the quality of Web surveys: the Checklist for Reporting Results of Internet E-Surveys (CHERRIES). J Med Internet Res. 2004 Sep 29;6(3):e34 [erratum in J Med Internet Res. 2012; 14(1): e8.]. Article available at [https://www.jmir.org/2004/3/e34](https://www.jmir.org/2004/3/e34/)/; erratum available <https://www.jmir.org/2012/1/e8/>. Copyright ©Gunther Eysenbach. Originally published in the [Journal of Medical Internet](http://www.jmir.org) Research, 29.9.2004 and 04.01.2012.

This is an open-access article distributed under the terms of the Creative Commons Attribution License (<https://creativecommons.org/licenses/by/2.0/>), which permits unrestricted use, distribution, and reproduction in any medium, provided the original work, first published in the Journal of Medical Internet Research, is properly cited.

**Supplementary Table 4.** SPIROS 2025 Checklist: Recommended Items to address in the observational study protocol and related documents

| **Section / Item** | **Item Number** | | **Description** |
| --- | --- | --- | --- |
| **Part A: General information** | | | |
| Title | 1 | ☑ | Descriptive title, identifying study design in the title |
| Protocol version | 2 | None | Version or amendment number with date and summary of the changes |
| Protocol summary | 3 | ☑ | An informative and balanced summary of the study protocol |
| Sponsor and funder details | 4 | ☑ | Name of Sponsor and funder and types of financial, material, and other support |
| Conflict of interest statements | 5 | ☑ | Statement about any financial and other competing interests for principal or co-investigators for the overall study. |
| Investigators name | 6a | ☑ | Names of the principal and co-investigators |
| Affiliation of investigators | 6b | ☑ | Affiliated institutions of the investigators |
| Principal researcher/s contact detail | 6c | ☑ | Name, e-mail address, affiliation of principal researcher |
| **Part B: Introduction** | | | |
| Background of the study | 7a | ☑ | Description of research question and scientific background of the study |
| Review of prior research | 7b | ☑ | Summary of relevant existing research (published or unpublished) |
| Rationale of study | 7c | ☑ | Justification for conducting the study |
| Aim | 8a | ☑ | Broader aims and overall objective |
| Objective/s of the study | 8b | ☑ | Primary and secondary objective/s including any prespecified hypothesis (if applicable). |
|  | 8c | ☑ | Specify whether the intention is to (a) estimate causal effects,  (b) predict outcomes, or (c) simple description. |
| **Part C: Methods** | | | |
| Study design | 9a | ☑ | Description of study design (case control, cross-sectional or cohort) and type of study (retrospective cohort study, Prospective cohort study etc.) |
| Study setting | 9b | ☑ | Description of the study setting (e.g., community-based, hospital based) and detail of precise locations of the study sites. |
| Study schedule | 10a | ☑ | Description of the expected schedule of the study including relevant dates, expected periods of recruitment/survey, exposure, follow-up, and data collection. |
|  | 10b | ☑ | Figure (Study schematic/flow-chart) or table describing expected time frame for each step including trainings, data collection, follow-up, analysis and reporting etc. |

| **Section / Item** | **Item Number** | | **Description** |
| --- | --- | --- | --- |
| Sample size | 11 | ☑ | Estimation of minimum sample size required for the study with justifications including clinical and statistical assumptions supporting any sample size calculations. |
| Sampling procedure | 12 | ☑ | Detailed description of the sampling frame and sampling strategy (simple random, stratified random, cluster, systematic etc.) |
| **Participant selection** |  |  |  |
| Participant selection for cohort study | 13a | NA | Description of inclusion and exclusion criteria, and the source and methods of participant selection (exposed and unexposed). For matched cohort studies, give matching criteria and number of exposed and unexposed. |
| Participant selection for case-control study | 13b | NA | Description of inclusion and exclusion criteria, and the source and methods of case ascertainment and control selection.  Give the rationale for the choice of cases and controls. Give diagnostic criteria for identifying cases (if applicable). For matched case-control studies, give matching criteria and the number of controls per case. |
| Participant selection for cross-sectional study | 13c | ☑ | Description of the inclusion and exclusion criteria, and the source and methods of participant selection. |
| Variables | 14a | ☑ | Detailed description of all important baseline and outcome variables to be analyzed, exposures, predictors, potential confounders, and effect modifiers. Give diagnostic criteria, if applicable. |
| Data sources/measurement | 14b | ☑ | For each variable of interest, give sources of data and details of assessment /measurement methods. Describe comparability of assessment methods if there is more than  one group. |
| Data collection and management | 15a | ☑ | Plans for assessment and collection of outcomes, baseline, follow up and other study related data. |
|  | 15b | ☑ | Description of data collection methods e.g., online survey, Household survey, paper based or electronic data capture,  etc. |
|  | 15c | ☑ | Any related processes to promote data quality during data collection (e.g., duplicate measurements, training of assessors, validation method) |
|  | 15d | ☑ | Description of study instruments (e.g., questionnaires, data collection forms) along with their reliability and validity, if known. Reference to where data collection forms can be found, if not in the protocol. |
|  | 15e | ☑ | Plans for data entry, coding, security, and storage, including any related processes to promote data quality (e.g., electronic data capture, double data entry; range checks for data values, random cross-checking of electronic data with the source documents). |

| **Section / Item** | **Item Number** | | **Description** |
| --- | --- | --- | --- |
|  | 15f | ☑ | Reference to where details of data management procedures can be found, if not in the protocol. |
| Blinding procedure (if blinded study) | 16 | NA | Description of blinding procedure (if applicable) reporting Who will be blinded (e.g., investigator blinded for disease status when measuring exposure in case-control study) and methods to ensure blinding and unmasking of blinding if  required. |
| Potential bias | 17 | ☑ | Description of any potential biases and plan to minimize those potential sources of biases. |
| Statistical analysis plan | 18 | ☑ | Detailed description of methods for analyzing and presenting primary/secondary outcomes and any additional analysis (e.g., analyses of subgroups and interactions, and sensitivity analyses). Give reference to the where other details of the statistical analysis plan can be found, if not in the protocol. |
| Handling of missing data | 19 | NA | Detailed description of methods to handle missing data (e.g., multiple imputation). |
| Handling of withdrawals and lost to follow up | 20a | NA | Detailed description of the procedures to be followed when a participant ceases participation in the study prematurely or is lost to follow up |
| Replacements | 20b | NA | Plans and methods of the replacement or substitution of withdrawn participants. |
| Outcome | 21 | ☑ | Definition and description of all primary, secondary and other outcomes. |
| Data confidentiality statement | 22 | ☑ | A detailed description of process to ensure data confidentiality. |
| Follow up | 23 | NA | A detailed plan of follow up including schedule and methods (telephonic, house based, hospital based etc.) of follow up. |
| Plan of study monitoring | 24 | NA | Description of plan for study monitoring and whether the monitoring will be independent from investigators or  sponsors. |
| Training of surveyors/data collectors | 25 | NA | Description of how investigators and surveyors will be trained to conduct the research activity. |
| Quality assurance | 26 | ☑ | Plan of quality assurance. back-checking data collection. |
| **Part D: Ethical consideration** | | | |
| Ethical approval | 27a | ☑ | Plan for seeking ethics approval from ethics committees/institutional review boards. If known, give name of ethical committees. |
|  | 27b | NA | If ethics approval will not be sought, give justification. |
| Consent and assent | 28a | ☑ | Description of who will obtain informed consent or assent from potential study participants or authorized surrogates, and how (e.g., written informed consent, verbal consent, video/audio recording of consent procedure etc.) |
|  | 28b | ☑ | Give reason if consent or assent not sought. |

| **Section / Item** | **Item Number** | | **Description** |
| --- | --- | --- | --- |
|  | 28c | NA | Give reference to where informed consent forms and  applicable translations plan can be found, if not in the protocol. |
| Risk/harm to participants | 29a | ☑ | A detailed description of potential risks or harms to study participants. |
|  | 29b | ☑ | Plans for collecting, assessing, reporting, and managing any study procedures related adverse events (e.g., adverse events due to blood collection) and other unintended effects of study conduct (e.g., risk to breach confidential and sensitive information of participants) |
|  | 29c | ☑ | Give a statement about whether data will be anonymous, pseudonymized, or can be directly linked to participants. |
|  | 29d | ☑ | Description of any plan for giving Incentives to the  participants |
| Adverse event and serious adverse event reporting | 30 | NA | Outline how adverse events and serious adverse events information will be collected and reported. |
| Involvement of patient/participant representatives in protocol development | 31 | ☑ | Patient and Public Involvement (PPI) statement including how patients or participants involved in the planning of the study. Give statement, if there is no plan to involve of patient/participants and public in designing or any phase of the study |
| **Part E. Reporting and dissemination** | | | |
| Dissemination/ publication plan | 32a | NA | Plans for investigators and sponsor to communicate study results to ethical review boards, participants, key stake holders, the public, and other relevant groups. |
|  | 32b | ☑ | Methods to communicate findings (e.g., via publication (open access or closed access), reporting in results databases, or other data-sharing arrangements), including any publication restrictions. |
|  | 32c | ☑ | Define authorship eligibility guidelines (e.g., ICMJE recommendations) |
| **Part F: Others** | | | |
| Whether Artificial Intelligence (AI) assisted technology was used in writing the protocol | 33 | ☑ | Disclose whether authors used artificial intelligence (AI)-assisted technologies in the production of protocol (e.g., chatbots) or there is planning to use artificial intelligence (AI)- assisted technologies in the production of manuscript or study reports. |
|  | 34 | ☑ | Give the name of AI tools (such as ChatGPT). Include a statement if authors did or did not review and edited the content created by AI-assisted technologies |
| References | 35 | ☑ | A complete list of references cited in protocol. |
| Funding | 36 | ☑ | Source of any funding for the study and the role of the funders for the study |
| Open science | 37a | ☑ | **Registration of observational study:** Study identifier and  registry name (e.g., open science framework, |

| **Section / Item** | **Item Number** | | **Description** |
| --- | --- | --- | --- |
|  |  |  | ClinicalTrials.gov, ICTRP or any other national or international  study registry platform). If not yet registered, name of intended registry. |
|  | 37b | ☑ | **Data sharing:** Plans, if any, for granting public access to the  (1) full protocol and amendments, (2) participant-level data set, (3) Statistical analysis plan, (4) statistical codes and other study material (e.g., case report forms, study questionnaires and Informed consent forms). Give reference to where these documents can be found, if not included as annex in the protocol. |

**REFERENCES**

[1] Gagnier JJ, de Arruda GT, Terwee CB, Mokkink LB. Consensus group. COSMIN reporting guideline for studies on measurement properties of patient reported outcome measures. Quality of Life Research. 2025, 34(7): 1901–1911. <https://doi.org/10.1007/s11136-025-03950-x>.

[2] López-Cepero AA, Spruill T, Suglia SF, Lewis TT, Mazzitelli N, Pérez CM, Rosal MC. Shift-and-persist strategies as a potential protective factor against symptoms of psychological distress among young adults in Puerto Rico. Social Psychiatry and Psychiatric Epidemiology. 2024, 59(8): 1357–1365. <https://doi.org/10.1007/s00127-023-02601-1>.

[3] Peduzzi P, Concato J, Kemper E, Holford TR, Feinstein AR. A simulation study of the number of events per variable in logistic regression analysis. Journal of Clinical Epidemiology. 1996, 49(12): 1373–1379. [https://doi.org/10.1016/S0895-4356(96)00236-3](https://doi.org/10.1016/s0895-4356(96)00236-3).

[4] Heinze G, Schemper M. A solution to the problem of separation in logistic regression. Statistics in Medicine. 2002, 21(16): 2409–2419. <https://doi.org/10.1002/sim.1047>.

[5] Preacher KJ, Rucker DD, Hayes AF. Addressing moderated mediation hypotheses: Theory, methods, and prescriptions. Multivariate Behavioral Research. 2007, 42(1): 185–227. <https://doi.org/10.1080/00273170701341316>.

[6] Asparouhov T, Muthén B. Auxiliary variables in mixture modeling: Three-step approaches using Mplus. Structural Equation Modeling: A Multidisciplinary Journal. 2014, 21(3): 329–341. <https://doi.org/10.1080/10705511.2014.915181>.

[7] Vermunt JK. Latent class modeling with covariates: Two improved three-step approaches. Political Analysis. 2010, 18(4): 450–469. <https://doi.org/10.1093/pan/mpq025>.

[8] Laursen BP, Hoff E. Person-centered and variable-centered approaches to longitudinal data. Merrill-Palmer Quarterly. 2006, 52(3): 377–389. <https://doi.org/10.1353/mpq.2006.0029>.

[9] Seol J, Iwagami M, Yanagisawa M. Association of sleep patterns assessed by a smartphone application with work productivity loss among Japanese employees. NPJ Digital Medicine. 2025, 8(1): 751. <https://doi.org/10.1038/s41746-025-02155-3>.

[10] McInnes L, Healy J, Melville J. UMAP: Uniform manifold approximation and projection for dimension reduction. 2018. Available at: <https://doi.org/10.48550/arxiv.1802.03426> (Accessed: 1 February 2026).

[11] Hassan BA, Tayfor NB, Hassan AA, Ahmed AM, Rashid TA, Abdalla NN. From A-to-Z review of clustering validation indices. Neurocomputing. 2024, 601: 128198. <https://doi.org/10.1016/j.neucom.2024.128198>.

[12] Ward MK, Meade AW. Dealing with careless responding in survey data: Prevention, identification, and recommended best practices. Annual Review of Psychology. 2023, 74(1): 577–596. <https://doi.org/10.1146/annurev-psych-040422-045007>.

[13] Wilkinson MD, Dumontier M, Aalbersberg IJ, Appleton G, Axton M, Baak A, *et al.* The FAIR guiding principles for scientific data management and stewardship. Scientific Data. 2016, 3: 160018. <https://doi.org/10.1038/sdata.2016.18>.
